# Assessing local resilience to typhoon disasters: A case study in Nansha, Guangzhou

**DOI:** 10.1371/journal.pone.0190701

**Published:** 2018-03-09

**Authors:** Jinglu Song, Bo Huang, Rongrong Li

**Affiliations:** 1 Department of Geography and Resource Management, The Chinese University of Hong Kong, Shatin, New Territories, Hong Kong; 2 Institute of Space and Earth Information Science, The Chinese University of Hong Kong, Shatin, New Territories, Hong Kong; 3 Shenzhen Research Institute, The Chinese University of Hong Kong, Shenzhen, China; Universidade de Aveiro, PORTUGAL

## Abstract

Building communities’ resilience to natural weather hazards requires the appropriate assessment of such capabilities. The resilience of a community is affected not only by social, economic, and infrastructural factors but also by natural factors (including both site characteristics and the intensity and frequency of events). To date, studies of natural factors have tended to draw on annual censuses and to use aggregated data, thus allowing only a limited understanding of site-specific hot or cold spots of resilience. To improve this situation, we carried out a comprehensive assessment of resilience to typhoon disasters in Nansha district, Guangzhou, China. We measured disaster resilience on 1×1-km grid units with respect to socioeconomic and infrastructural dimensions using a set of variables and also estimated natural factors in a detailed manner with a meteorological modeling tool, the Weather Research and Forecast model. We selected typhoon samples over the past 10 years, simulated the maximum typhoon-borne strong winds and precipitation of each sample, and predicted the wind speed and precipitation volume at the 100-year return-level on the basis of extreme value analysis. As a result, a composite resilience index was devised by combining factors in different domains using factor analysis coupled with the analytic hierarchy process. Resilience mapping using this composite resilience index allows local governments and planners to identify potential hot or cold spots of resilience and the dominant factors in particular locations, thereby assisting them in making more rational site-specific measures to improve local resilience to future typhoon disasters.

## Introduction

Coastal areas are facing greater and greater threats from natural disturbances, from both long-term phenomena (such as rising sea levels and climate change) and short-term weather events (such as typhoons, tsunamis, and earthquakes). However, because short-term events are characterized by sudden appearances, uncertain paths, and destructive power, when they occur, coastal regions are likely to experience many casualties and great losses of property. The 2013 Typhoon Usagi is one example of such a short-term natural disturbance that caused enormous damage in the southern coastal area of China (2014 Yearbook of Meteorological Disasters in China). The direct damage caused by these disasters normally includes the destruction of infrastructure, property loss, injuries, and casualties, and the indirect effects include socioeconomic instability and high recovery costs [1, 2]. Therefore, in disaster risk management, the means of reducing vulnerability and accelerating the recovery process of coastal regions—in other words, the improvement of disaster resilience—is a multifaceted challenge for policy makers. Given the greater frequency and destructiveness of coastal events in recent years, this issue has attracted increasing attention [3–6].

Although the literature emphasizes the importance of disaster resilience research and management, no consensus has been reached on a clear definition of disaster resilience [7, 8]. The concept has been taken in two main directions: some researchers have considered disaster resilience to be the product of a set of related items such as vulnerability and recovery capability [9–18], whereas others have considered it to be a multifaceted concept that involves indicators as proxies for resilience in a set of sub-dimensions, such as social resilience, economic resilience, and infrastructural resilience [1, 19–21]. For the first direction, it could clarify the context of resilience and make it easily understood from a qualitative perspective, whereas conceptualization in the second direction could help to build a more applicable framework for resilience assessment. In fact, conceptualizations in the second direction usually suffer from a subjective selection of indicators in different dimensions. This problem could be mitigated in the context of resilience (e.g., vulnerability and recovery capability) as defined in the first direction. Consequently, an integrated definition is proposed in this study, in which disaster resilience is conceptualized as the ability of an urban system and its subcomponents (e.g., social, economic, infrastructural, and natural components) to withstand or rapidly return to desired functions in the face of extreme events. Therefore, indicators used to manifest the multifaceted nature of resilience in the second direction (e.g., social, economic, infrastructural, and natural components) should fully capture the characteristics of elements mentioned in the first direction (vulnerability and adaptive capacity) for content validity. Content validity is a guiding principle in initial development of a measure to ensure that all domains of the concept to be measured are included in the measure [22, 23]. As exposure is defined as one component of vulnerability, which is determined to a large extent by the intensity and frequency of a natural disaster, factors related to such characteristics of exposure should also be considered in indicator selection. In other words, factors in the natural dimension should also be included in addition to those in the social, economic, and infrastructural domains if one wishes to conceptualize resilience in light of the second direction.

In view of the different interpretations of disaster resilience, researchers have proposed a variety of frameworks to measure it [7, 16, 24–28], within which sets of models or index systems are mostly used, such as the Disaster Resilience of Place (DROP) model [29], the Climate Disaster Resilience Index (CDRI) [30], the Resilience Capacity Index [31], the Baseline Resilience Indicator for Community (BRIC) [21], and the Resilience Inference Measurement (RIM) index [28]. The natural factors in these models or index systems refer mostly to site-specific characteristics (e.g., loss of wetlands and the percentage of impervious surface in the DROP model). The exceptions are the CDRI and BRIC systems, in which the characteristics of natural disasters (e.g., the intensity and frequency of natural disasters in the CDRI) or their influences (e.g., flooding buffer in BRIC) are also considered.

Although a body of research has already taken account of natural factors (e.g., the intensity and frequency of natural disasters) when measuring disaster resilience, the collection of consistent high-quality data remains a challenge when exploring natural factors [7, 32]. To overcome the problem caused by this data shortage, a few studies have applied data derived from modeling results. A landmark report in which such an approach was used was proposed by Cutter et al. (2008) [20], who used data obtained via simulation and aggregated it at the community level to depict 100-year flooding zones and storm surge inundation zones when measuring coastal disaster resilience in New Jersey communities. In addition, success in the application of modeling outcomes was demonstrated by a set of existing vulnerability assessments, in which data were obtained by simulating the natural parameters of long-term phenomena such as climate change and rising sea levels [33, 34]. For the assessment of short-term disaster resilience, the aforementioned approaches could offer helpful inspiration for the quantification of natural factors such as wind speed and precipitation data for indexing local natural resilience to typhoon disasters.

The aim of this study was to develop a composite resilience index to assess disaster resilience by combining factors in different resilience domains. To achieve this purpose, we focus on some important issues in disaster resilience measurement, including the selection of resilience indicators, data acquisition for indexing natural factors, weight determination of each factor using factor analysis (FA) coupled with an analytic hierarchy process (AHP), and interpretation of the spatial differences in disaster resilience on the basis of the final assessment results. In addressing these issues, we attempt to assess disaster resilience in a case study of Nansha district, Guangzhou, China, where typhoons are common in summer. We first conduct simulation and prediction work to acquire indicators related to wind speed and precipitation in a high spatial resolution. We then incorporate the prediction results into microscale resilience assessment and construct a hierarchical framework for a composite resilience index using weights from both the objective and subjective perspectives. According to the assessment results, we then elucidate the spatial heterogeneity of typhoon disaster resilience and its site-specific dominant factors within the study area. Finally, we conclude with a discussion of our findings and the implications for planning practice and natural hazard mitigation.

## Study area

**Fig 1:**
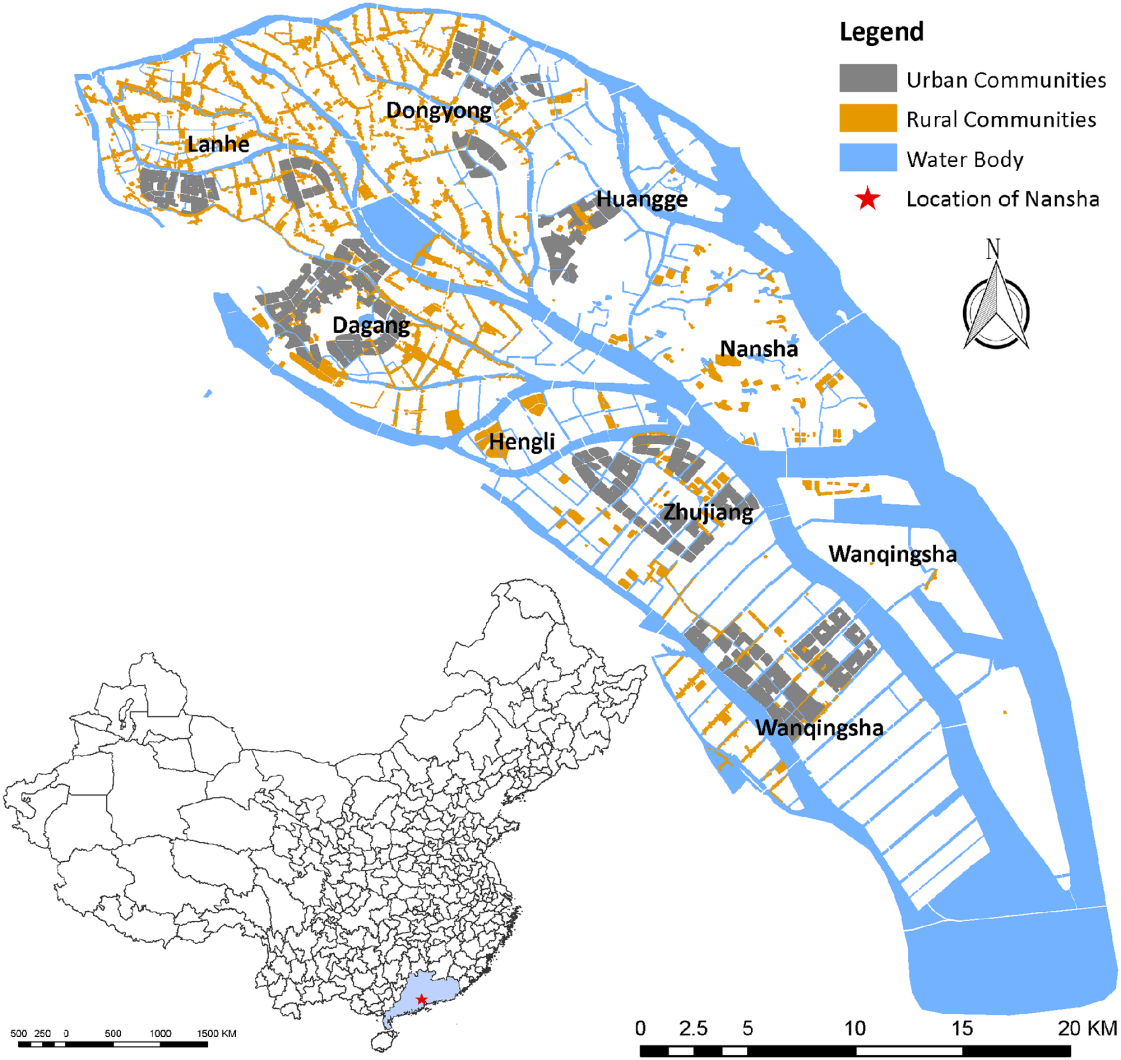
Location map of Nansha district, Guangdong province, China. Nansha district consists of two sub-districts and six towns. The locations of urban and rural communities are based on the Comprehensive Planning of Nansha New City (2012) [35]. The map was generated using the free and open source software QGIS version 2.18 (http://www.qgis.org/en/site/about/index.html).

Nansha District in Guangzhou is located on the southeastern coast of mainland China (Fig 1). The district has 25.5 km of shoreline, a low average elevation (about 2 m), and a subtropical monsoon climate, which make it susceptible to the effects of typhoons that originate in the northwestern Pacific. This district has undergone significant population growth and economic development over the past few decades in accordance with China’s 12^th^ Five-Year Plan (2011–2015), and future development will continue due to its location connecting Guangzhou, Hong Kong, and Macao. Therefore, there is an urgent demand for the improvement of disaster resilience in this area.

## Modeling wind speed and precipitation of typhoons

Nansha lies on the southeast coast of China, where it is exposed to typhoon influences all year, especially those from the northwest Pacific Ocean. Therefore, for Nansha, resilience to disasters mostly takes the form of resilience to typhoon-caused disruptions, such as gale damage and flooding. In this case, the characteristics of natural hazards in the model focus on the severity and frequency of typhoons and the damage they cause. Indicators that represent the extreme wind speeds and precipitation of typhoons could enable better resilience assessment.

**Fig 2:**
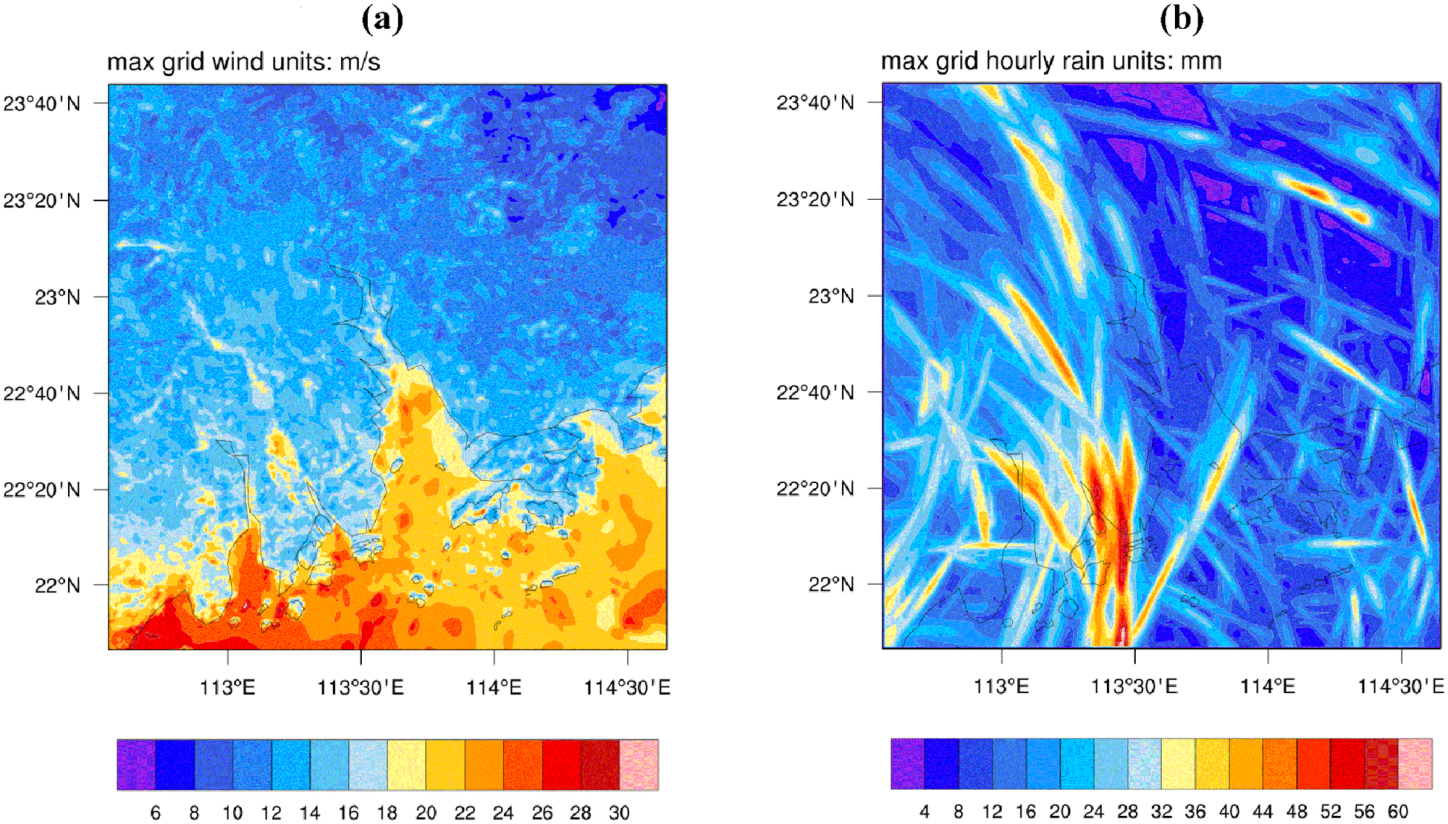
Sample results of typhoon Utor. (a) maximum grid wind distribution (unit: m/s); (b) maximum grid hourly precipitation (unit: mm/h); resolutions of both are 1 × 1 km. Wind speed modeled in this study is at 850 hpa height because wind speed at 850 hpa height is considered surface wind in meteorology and has the greatest effect on surface features, such as buildings and infrastructure. The map was generated using the free and open source software NCAR Command Language version 6.4.0 (2017) (http://dx.doi.org/10.5065/D6WD3XH5).

**Fig 3:**
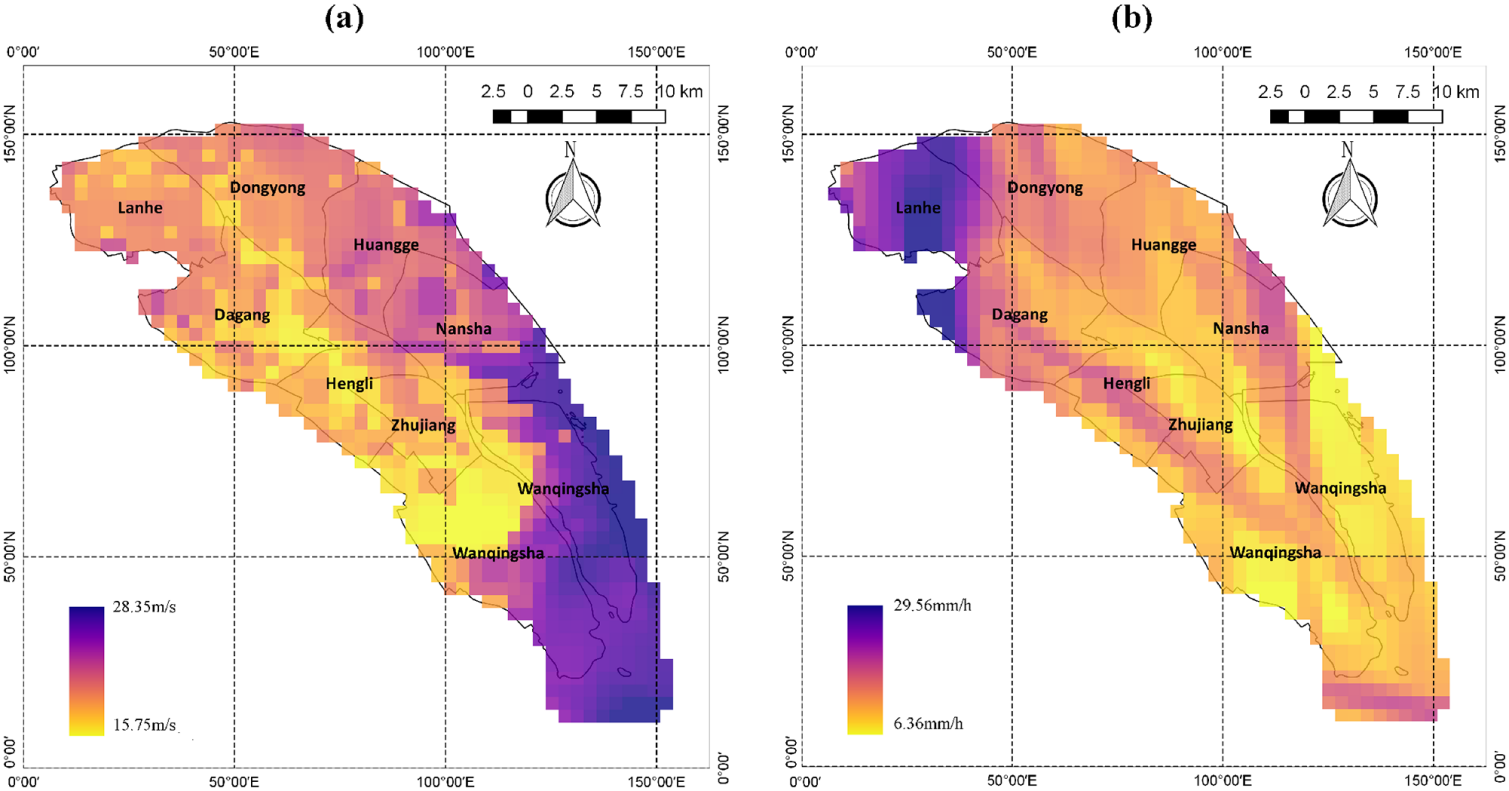
Modeling results for Utor in Nansha. (a) distribution of maximum wind speed (unit: m/s); (b) maximum precipitation (unit: mm/h) in each grid square in Nansha; both have resolution of 1 × 1 km. The map was generated using the free and open source software QGIS version 2.18 (http://www.qgis.org/en/site/about/index.html).

Because observational data, which have raw resolution in both spatial (tens of kilometers) and temporal (up to 6-hour intervals) dimensions, are not sufficiently detailed to provide the historical maximum wind speed and hourly precipitation data at the high resolution required in this study, we made use of modeling results. Twenty-four samples were selected from typhoons during the past decade according to Nansha’s annual meteorological reports from 2010 to 2013 and records published on the Hong Kong Observation website. Detailed information about the 24 typhoon samples is listed in Table 1. The maximum wind speed and maximum hourly precipitation at each analysis unit (grid square at a 1×1-km resolution) were simulated for each sample using the Weather Research Forecast (WRF) model. The modeling results of Typhoon Utor are shown as examples in Figs 2 and 3. The physical schemes used in the WRF model and the validation of simulation results can be found in S1 Text.

**Table 1:** Information on 24 selected typhoon samples.

Typhoon Code	Name	Duration of Modeling
1319	Usagi	2013/9/20/00–2013/9/23/00
1311	Utor	2013/8/12/00–2013/8/15/00
1309	Jebi	2013/7/31/12–2013/8/3/12
1306	Rumbia	2013/6/29/12–2013/7/2/12
1213	Kai-Tak	2012/8/15/00–2012/8/18/00
1206	Doksuri	2012/6/27/00–2012/6/30/00
1117	Nesat	2011/9/27/00–2011/9/30/00
1108	Nockter	2011/7/27/12–2011/7/30/12
1011	Fanapi	2010/9/18/00–2010/9/21/00
1010	Meranti	2010/9/8/00–2010/9/11/00
1006	Lionrock	2010/8/31/00–2010/9/3/00
1003	Chanthu	2010/7/20/00–2010/7/23/00
1002	Conson	2010/7/14/00–2010/7/17/00
0915	Koppu	2009/9/13/00–2009/9/15/12
0906	Molave	2009/7/16/00–2009/7/19/00
0904	Nangka	2009/6/24/00–2009/6/27/00
0817	Higos	2008/10/3/00–2008/10/6/00
0814	Hagupit	2008/9/21/12–2008/9/24/12
0812	Nuri	2008/8/19/12–2008/8/23/00
0714	Francisco	2007/9/23/00–2007/9/25/00
0606	Prapirou	2006/8/1/00–2006/8/4/00
0601	Chanchu	2006/5/15/00–2006/5/18/00
0518	Damrey	2005/9/23/00–2005/9/26/00
0418	Aere	2004/8/24/12–2004/8/27/12

**Note**: The typhoon code is determined by the year (first two digits) and the order (the last two digits) that the typhoon sample occurred. To reduce simulation error on the typhoon track, each typhoon sample was tracked 2–3 days before and 1–2 days after its landfall time.

**Fig 4:**
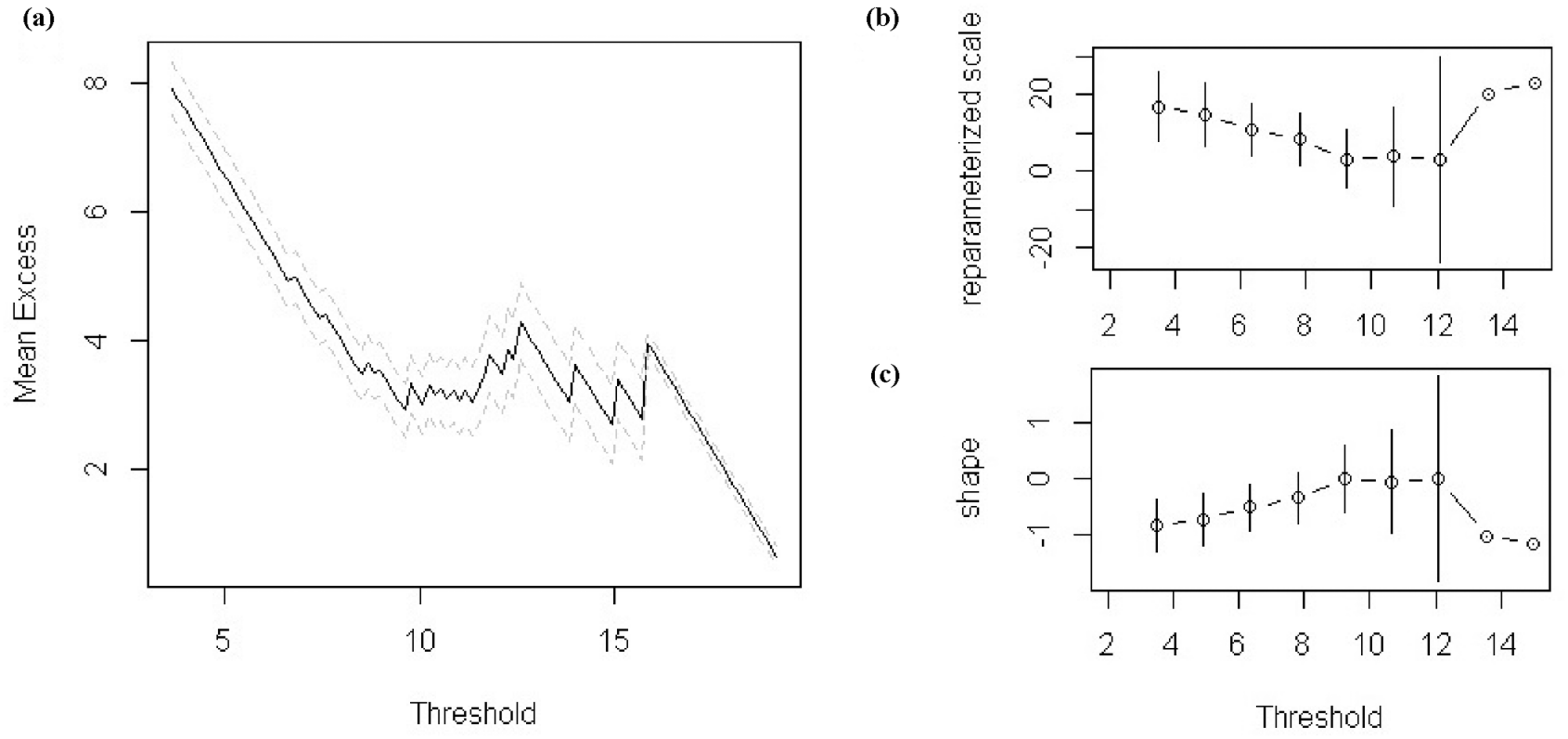
Threshold value and parameter estimation of typhoon maximum wind speed in sample grid. (a) Mean residual life plots; (b) Re-parameterized scale parameter; (c) Shape parameter. Approximate straight line in (a) from 9 to 12 implies that suitable threshold value should be around 9, and similar trends of two parameters are also presented in (b) and (c).

To construct suitable indicators to describe the frequency and severity of future typhoon disasters, we then automated the extreme value model of Gilleland and Katz (2011) [36] to predict the wind speeds and precipitation of typhoons at the 100-year return level for each grid square at a 1×1-km resolution. This approach assumes that historical records follow a generalized Pareto distribution above a threshold value, and the threshold value for this model is determined by the mean residual life plot method [37]. To determine the threshold value, we delineated the mean residual life curve of each grid on the basis of its simulated maximum values (maximum wind speed or maximum 1-hour precipitation of all typhoon samples). We then detected the minimum value with little fluctuation in the mean residual life curve as the break and chose this minimum value as the suitable threshold value. We then estimated the other two parameters needed for further extreme value analysis, the shape and re-parameterized scale, by maximum likelihood estimation. Detailed operations of this estimation method are described elsewhere [38–40]. To make it clear, the results of one sample grid were taken as an example and are presented in Fig 4. Based on the derived three parameters, we implemented the extreme value model to predict the maximum values of wind speed and 1-hour precipitation of typhoons at the 100-year return level in R using functions in the *exRemes 2*.*0* package [41].

## Building a composite resilience index with modeling outcomes

In addition to the modeled characteristics of a typhoon disaster, to construct a composite resilience index, a number of variables should be collected as proxy measures to represent the multidimensional nature of disaster resilience. For a small-scale study, like the resilience assessment of Nansha District in this study, parameterization of resilience characteristics to disaster requires determination of the infrastructural, social, and economic factors of many sites and the corresponding geographic location data [42]. This not only involves more difficulty in the collection of data, but also implies that the resilience framework varies in different areas for which localized indicators are essential for resilience management. Thanks to data support from the local government, we collected and constructed site-specific indicators to represent the different components of resilience based on the disaster resilience literature. The indicators selected as proxies for typhoon disaster resilience are listed in Table 2.

**Table 2:** Selected indicators for resilience assessment.

Variable	Measures	Unit	Contribution	Justification	Source
Access to fire stations	Distance to nearest fire station	km	negative	[27]	FDNS
Access to public shelters	Distance to nearest public shelters	km	negative	[27]	FDNS
Access to sanitation	Distance to nearest sanitation	km	negative	[21]	FDNS
Alternative capacity of fire stations	Number of fire stations within 5-km radius service area along transport network	Unit	positive	[27]	FDNS
Demand of electricity	Demand of power supply per square kilometers	W/s	negative	[27]	FDNS
Demand of water supply	Demand of water supply per square kilometers	m3/s	negative	[27]	FDNS
Density of commercial infrastructure	Kernel density of commercial buildings	-	negative	[21]	FDNS
Density of illiterate persons	Number of illiterates per square kilometers	persons/km2	negative	[27]	CS
Density of migrant population	Number of migrant population per square kilometers	persons/km2	negative	[44]	CS
Density of population over 65	Number of people over 65 years old per square kilometers	persons/km2	negative	[7]	CS
Density of population under 15	Number of people under 15 years old per square kilometers	persons/km2	negative	[7]	CS
Density of transportation network	Length of transport network per square kilometers	km/km2	positive	[7]	FDNS
Density of urban green space	Kernel density of urban green space	-	positive	[27]	FDNS
Drainage density	Kernel density of drainage network	-	positive	[45]	FDNS
Engineering geological quality	Grade of engineering geological quality	-	negative	[27]	FDNS
Health access	Distance to nearest health facility (e.g., health post, hospital, clinic)	km	negative	[21]	FDNS
Population density	Number of people per square kilometers	persons/km2	negative	[46]	PGC [47]
Price of land	Price of land (RMB) per square meters	RMB/m2	negative	[27]	FDNS
Sheltering capacity	% open space per square kilometers	%	positive	[46]	FDNS
Slope	Degree of slope	degree (°)	negative	[48]	GDEMV2
Surface elevation	Height in digital elevation model (DEM)	m	negative	[31]	GDEMV2
Transport accessibility	Distance to nearest transport network	km	negative	[27]	FDNS
Typhoon precipitation	Extreme value of precipitation at 100-year return level	mm/h	negative	[27]	MR
Typhoon wind speed	Extreme value of wind speed at 100-year return level	m/s	negative	[27]	MR
Unemployment rate	Ratio of unemployed people over total population	-	negative	[7]	CS

Note: Indicators are sorted ascendingly. The 2010 China township population census data (CS) was obtained from Statistics Bureau of Guangzhou Municipality (http://www.gzstats.gov.cn/pchb/dlcrkpc/). The 1×1-km population grid dataset of China in 2010 (PGC) was obtained from Chinese Academy of Sciences (http://www.geodoi.ac.cn/WebEn/Default.aspx). The fundamental database of Nansha District (FDNS) was obtained from the Nansha District Planning Bureau of Guangzhou (http://www.gzns.gov.cn/nssj/). The modeling results (MR) were achieved through WRF simulation of typhoon samples and extreme value analysis. The DEM and slope data (GDEMV2) were derived from the ASTER GDEM dataset published in 2009 (https://earthexplorer.usgs.gov/).

Because no consensus has been reached on a definitive set of indicators for resilience measurement, the selection of indicators and the weight determination for each indicator are subjective to some extent. Because a composite index is a mathematical combination of individual indicators, this subjectivity would affect the reliability (internal consistency) of composite indicators during a combination process [43]. To solve this problem, this study includes a series of multivariate analyses to discriminate potentially relevant data from non-relevant data for a parsimonious set of metrics, while at the same time determining the weight of each indicator from both subjective and objective perspectives. In doing so, the multivariate analyses are divided into three steps: 1) to standardize the raw data via a Min-Max rescaling scheme; 2) to determine the weights of standardized variables via FA and derive a minimum number of upper indicators; and 3) to linearly combine the derived factors with weights assigned through the AHP and generate the final composite index of resilience.

Because the values of raw data have different dimensions and contributions to system resilience, in the first step, all raw data for the 688 grids were standardized with a Min-Max rescaling scheme. Different functions are used with respect to the known influences of indicators on resilience, based on previous studies of factors that increase (1) or decrease resilience (2). After the Min-Max rescaling procedure, each variable is transformed into an identical range between 0 and 1, where a value of 0 is the worst rank for an indicator score and 1 is the best rank.

$$\text{Positive}\;\text{effect}\;\text{that}\;\text{strengthens}\;\text{the}\;\text{result}:\;r_{i,j}=\frac{x_{i,j}-x_{min}}{x_{max}-x_{min}}$$ (1)

$$\text{Negative}\;\text{effect}\;\text{that}\;\text{weakens}\;\text{the}\;\text{result}:\;r_{i,j}=\frac{x_{max}-x_{i,j}}{x_{max}-x_{min}}$$ (2)

in which

*r*_*i*,*j*_: the standardized value of i^th^ object in the j^th^ indicator;

*x*_*i*,*j*_: the original value of i^th^ object in the j^th^ indicator;

*x*_*max*_: the maximum value in the original j^th^ indicator;

*x*_*min*_: the minimum value in the original j^th^ indicator.

After standardization, the procedures focus on determining the weights of these variables for a composite index of resilience. Because a number of indicators are at the bottom level and significant high correlations are detected between these variables, conventional approaches, such as expert score, which subjectively determine the weights of indicators, probably ignore or underrate the influences from the high degrees of correlation between individual variables. To solve this problem, FA is conducted on the standardized z-scores related to 26 grid-level variables in the second step. The FA procedure could increase the differences between the components, while at the same time making variable members of each factor exhibit similar variation across the study area. After comparing extraction results based on different rotation approaches, an equamax rotation was selected as the optimal rotation method because its extracted factors are more explainable with respect to previous findings. Via the aforementioned procedures in FA, a well-defined nested structure of the index is constructed from both theoretical and statistical perspectives [49]. The component loading for an individual variable is considered to be significant at 0.4 or above. Because the adjustment has been made to the indicators’ directionality during the Min-Max rescaling procedure, each of the extracted factors is expected to have a positive directionality and is believed to increase resilience.

**Fig 5:**
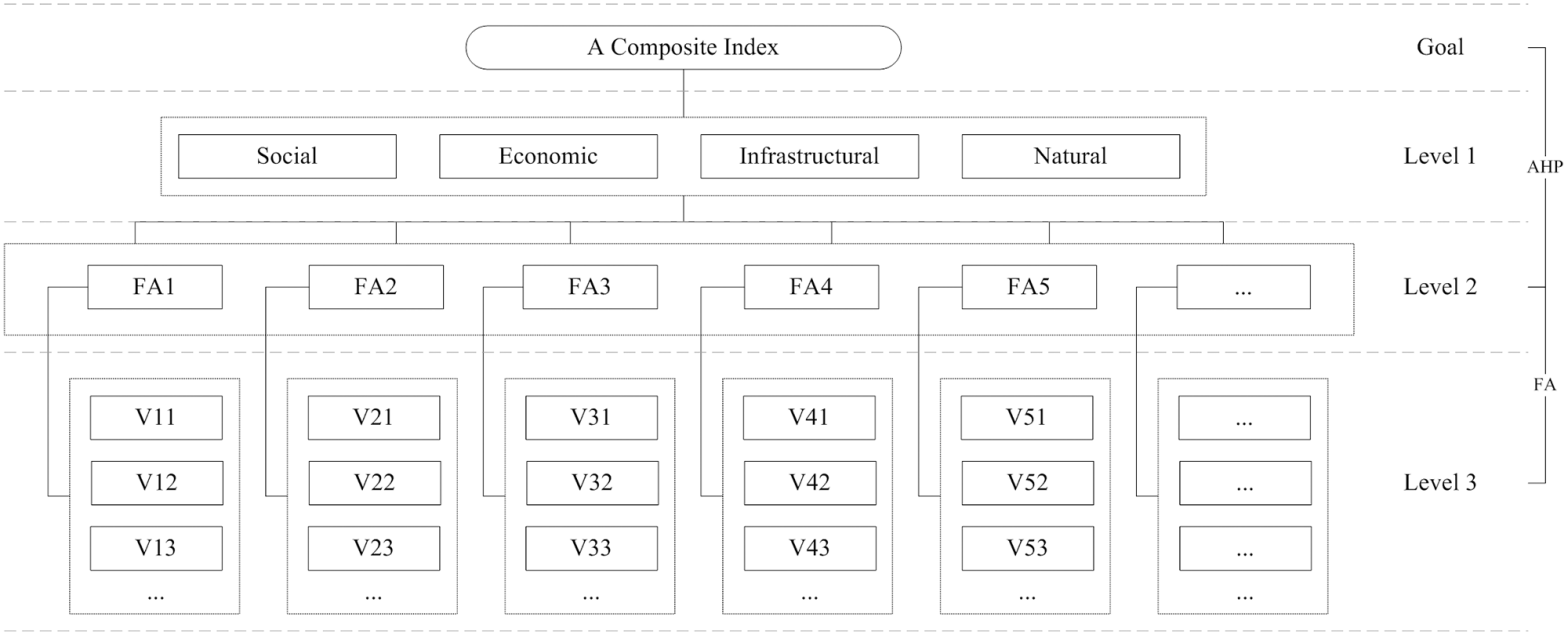
Hierarchical structure of composite resilience index in Nansha district.

The third step attempts to assign a weight to each factor score and combine the extracted factors into a composite resilience index. In some studies, the factor scores are equally weighted [50] or weighted by the percentage of variance explained [51]. This is considered appropriate due to the lack of justification for explicit weights and the lack of well-established relationships between variables [50]. However, this study, intended for local governments and planners, serves as a substitute for the decision-making task in their future resilience planning. The weights of factors should be determined according to their importance to the real case study rather than just reliability from a statistical perspective. Therefore, AHP is conducted as the third step to assign a weight to each factor score. The AHP approach is considered to be an efficient and flexible framework based on psychology and mathematics and is thus an ideal subjective weighting method. By analyzing the associations between factors with respect to existing studies, this approach builds up a hierarchical structure of the resilience index in Nansha District, including the goal and the first-level, second-level, and third-level indicators (Fig 5). The third level contains variables subordinate to the factors extracted in the second level. The first level includes indices that represent resilience in different dimensions based on the judgements of previous studies. The goal level represents the composite resilience index, which serves as the objective decision in this study. To determine the relative weight of each indicator at the same level, the analytic hierarchy process (AHP) [52] was used in this study. A comparative scale was adopted, where 1 means that the factors are equally important and a number larger than 1 means that a factor is more important than another. The comparison process was performed separately for each pair of factors under the same dimension at the lower level and for each pair of indices at the upper level. By means of the pair comparisons, judgement matrices were established for the opinions of local government and planners. A weighting vector was determined on the basis of these matrices.

To explore the discordances between the pairwise comparisons and the reliability of the obtained weights, the AHP must compute a consistency ratio (CR) by comparing the consistency index (CI) with the appropriate number listed in Table 3, each of which is an average random consistency index (RI). The CI of a matrix of comparisons is derived by (λ_max_−n) / (n − 1), where λ_max_ represents the sum of the products between the sum of each column of the comparison matrix and the relative weights and n is the size of the matrix. The RI table (Table 3) was derived by Saaty (1987) [52] from a sample of 500 randomly generated matrices. In the AHP, the CR value must be about 0.10 or lower. If not, it is necessary to revise the subjective judgements.

**Table 3:** Random index (RI) values.

n	1	2	3	4	5	6	7	8	9	10
**RI**	0.00	0.00	0.58	0.90	1.12	1.24	1.32	1.41	1.45	1.49

After deriving a weighting vector based on accepted subjective judgement metrics, a composite resilience index could be calculated by linear combination with respect to the extracted indices and weight assigned hierarchically following the structure in Fig 5.

## Results and analysis

### Typhoon modeling

**Fig 6:**
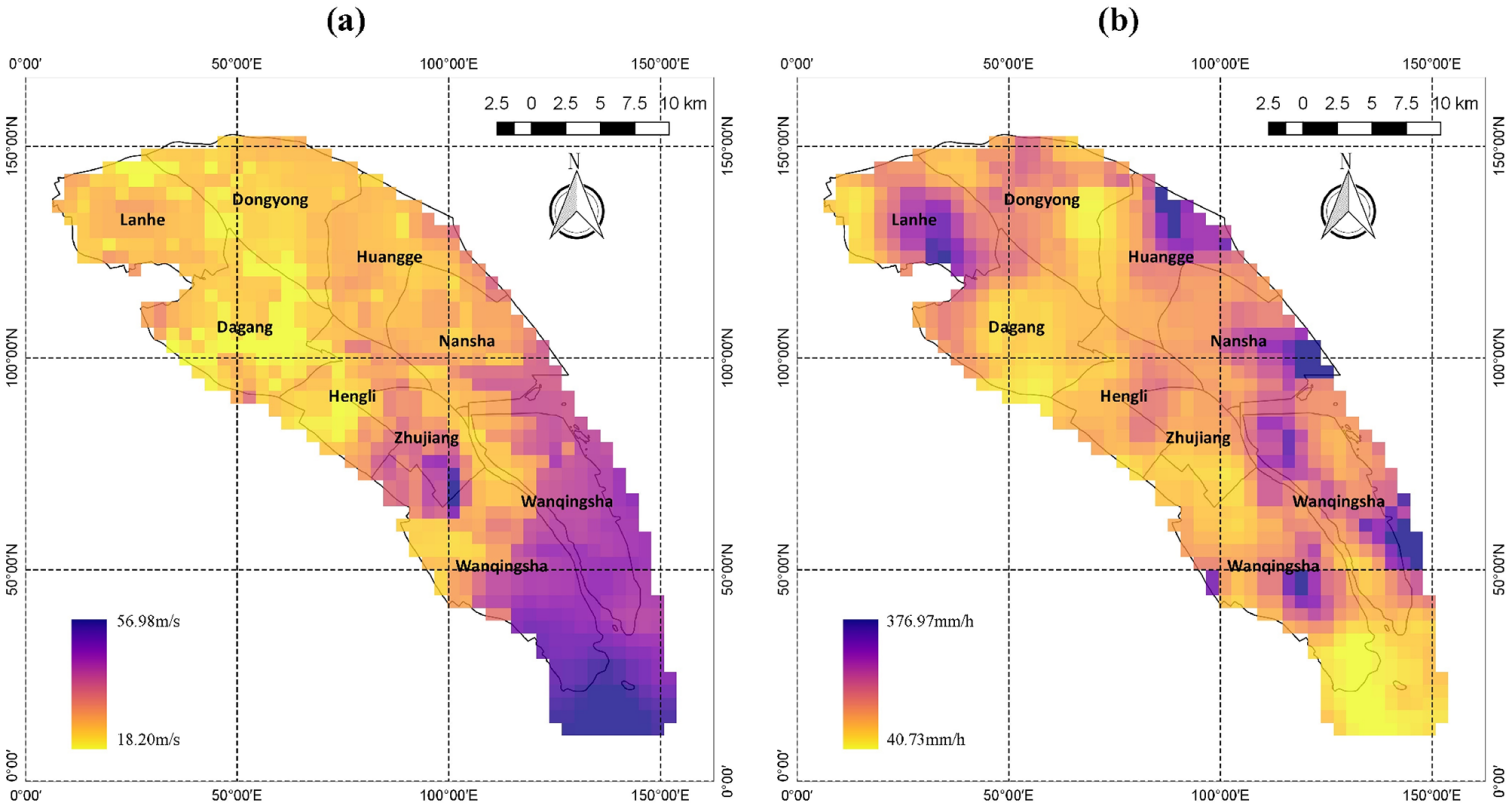
Results of extreme value analysis. (a) wind speed distribution (unit: m/s); (b) precipitation map (unit: mm/h); both are at 100-year return level. The map was generated using the free and open source software QGIS version 2.18 (http://www.qgis.org/en/site/about/index.html).

By means of extreme value analysis based on WRF simulations, the maps of maximum typhoon wind speed and maximum typhoon precipitation are shown in Fig 6. Both are at the 100-year return level with regard to local planning requirements. According to Fig 6a, the maximum typhoon wind speed exhibits a general decline from the southeast coast to the northwest, except for one hot spot in the southwest region. This is explainable because the coastal regions are considered to be typhoon-prone areas that see the highest typhoon wind speeds, and the wind strength would gradually weaken after the typhoon makes landfall. Higher predicted typhoon wind speeds indicate that these areas would suffer greater exposure to the damage caused by typhoon winds. With regard to Fig 6b, the maximum precipitation is not exhibited as regular distribution as extreme wind speed because typhoon precipitation is influenced not only by ground friction but also by other factors, such as tracks of typhoon eyes and cold air-masses.

**Fig 7:**
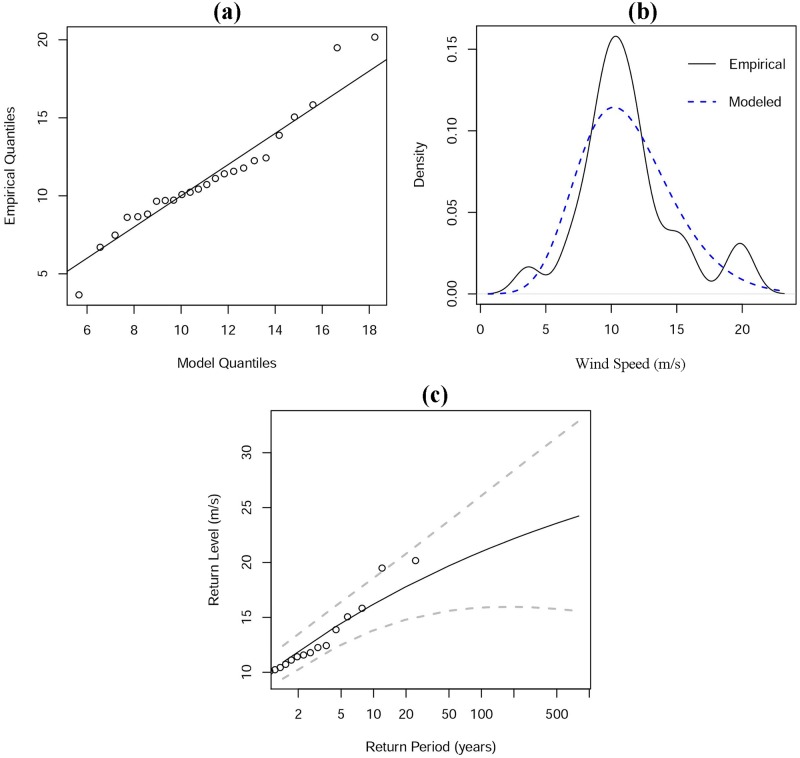
Diagnostics from the fitted generalized Pareto distribution function (modeled dataset) to the WRF-simulated maximum wind speeds (empirical dataset). (a) quantile plot, (b) density plot, and (c) return level plot.

In addition, to explore the fitness of predictions from a statistical perspective, we generate a set of diagnostics plots of each grid to using the corresponding functions in the *exRemes 2*.*0* package [41]. For a clear explanation, a sample grid is selected (S5 Fig) and the diagnostics of the sample grid are plotted in Fig 7, in which the WRF-simulated maximum wind speeds are labeled as the empirical dataset whereas a sample dataset drawn from the fitted GP df as the modeled dataset. In the plot of modeled quantiles against the empirical quantiles (Fig 7a), points approach the 45° line (y = x), indicating that the fitted GP df is in good agreement with the WRF simulation. In the density plot (Fig 7b), the empirical density curve approaches that of the modeled density curve, indicating that the fitted GP df fits well with the WRF-simulated value. Fig 7c describes the 100-year return level for wind speed, and its plots approach the central recurrence curve drawn from the fitted GP df and are almost within the 95% confidence interval, which implies a satisfactory estimation. Therefore, according to the diagrams of the fit diagnostics, the performance of the model’s predictions for the sample grid is acceptable. To extrapolate the model outcome from the sample to the population, we also performed a chi-square test. The p value was 0.2917, which is greater than 0.05 and implies an acceptable modeling result. After estimating the 100-year return levels for other grids in each typhoon sample, this model was able to predict the final distributions of extreme values of both wind speed and precipitation at the 100-year return level.

### The composite resilience index

The FA of 688 populated grids in Nansha District results in nine broad factors that explain 79.76% of the total variance (Table 4). The nine factors construct the second-level criteria in the hierarchical structure and at the same time are summarized in four up-level indices—social, economic, infrastructural, and natural components of resilience—based on previous results.

**Table 4:** Resilience components in first level, extracted factors in second level, and primary variables of each factor.

Component	Extracted Factors	Eigenvalue	% of variance	Primary variables	Loadings
Social	Demographics	4.112	16.447	Population density	0.959
				Density of population under 15	0.971
				Density of population over 65	0.933
				Density of illiterate persons	0.841
				Density of migrant population	0.751
Social	Community preparedness/service	2.655	10.62	Health access	0.73
				Access to sanitation	0.738
				Transport network density	0.529
				Transport accessibility	0.696
				Density of urban green space	0.479
Economic	Industry and asset exposure	2.462	9.849	Demand of electricity	0.842
				Demand of water supply	0.626
				Prices of land	0.895
Infrastructural	Response rapidness and capacity	2.227	8.907	Drainage density	0.526
				Access to fire stations	0.653
				Alternative capacity of fire stations	0.746
Economic	Economic stability and commercial infrastructure exposure	2.126	8.505	Unemployment rate	0.839
				Density of commercial infrastructure	0.781
Infrastructural	Sheltering access and capacity	1.924	7.695	Access to public shelters	0.868
				Sheltering capacity	0.773
Natural	Elevation and slope	1.744	6.975	Surface elevation	0.876
				Slope	0.895
Natural	Strong storm risk and landslide risk	1.461	5.843	Engineering geological quality	0.441
				Typhoon wind speed	0.917
Natural	Flooding risk	1.229	4.916	Typhoon precipitation	0.950

Note: These extracted nine factors could explain 79.76% of total variance based on FA with an equamax rotation. Because input variables have been transformed through Min-Max scheme, all factors will exhibit positive contribution to final assessment of disaster resilience.

The first component, social resilience, summarizes two derived factors: demographics and community preparedness/service. The demographic attributes of the analysis units could be connected to social capabilities, which suggest that regions with higher illiteracy rates and larger proportions of the elderly, children, and teenagers will likely exhibit less resilience than others [49, 53]. In the study area, such regions are mostly located in rural communities. Due to lower urbanization level, most rural communities lack education resources, resulting in higher illiteracy rates. The fewer employment opportunities enforce young agricultural labor force to move out, resulting in higher rates of emigration. On this condition, most people in rural communities have very limited knowledge of disasters and labor support for disaster response and rescue [27, 44]. The different conditions between urban communities and rural communities provide context and perspective for demographic attributes that are highlighted by FA due to their drastic variation across the study area. For the other factor, primary variables related to community preparedness/service are connected to health access, access to sanitation, transport capacity and accessibility and are used to measure how well the community functions before and after the disaster [54]. The uneven allocations of these disaster preparedness and relief facilities were also highlighted in FA and were represented by the derived community preparedness/service factor.

For the second component, economic resilience is designed to measure industry and assess exposure, economic stability, and commercial infrastructure exposure. The former factor is extracted to represent primary indicators related to demands for electricity and power and the price of land. With regard to vulnerability, as one important theme of resilience, a higher demand for electricity and power is usually related to greater industrial development and higher land prices, and the corresponding regions thus suffer greater exposure to extreme events and have greater difficulty recovering after a catastrophe. The other factor represents variables related to differences in employment and commercial establishment across the study area and indicates that the unemployment rate is associated with commercial establishment across the study area. Because a high rate of unemployment indicates less stability of livelihood support for recovery [20], whereas dense commercial infrastructure implies a higher risk of urban property loss and a greater recovery cost after the disaster [55], higher values of these two indicators collectively imply lower resilience in the study area.

For the third component, infrastructural resilience, two extracted factors are both related to variations based on response and rescue capabilities. Indicators related to critical infrastructure, such as drainage, fire stations, and shelters are used to represent response rapidness and redundancy [20, 49]. The variance in these indicators could cause differences in coping capability during the disaster and the recovery duration and cost afterward.

The last three factors reflect variations related to natural resilience across the study area. Under this component, the first extracted factor suggests that areas with a high elevation have greater slope, which is reasonable because most parts of Nansha District are flat, except for a set of small hills. Areas with high elevations and steep slopes have a high landslide risk during and after typhoon disasters and thus have lower disaster resilience. The second factor suggests that high wind speed varies are in accordance with engineering geological quality. With regard to the study area, areas that have a greater historical record of landslide or land subsidence are located around mountainous regions and coastal areas, where are have a high risk of strong storm damage. The last factor, typhoon precipitation, is used to represent the flooding risk coupled with drainage density, which represents the runoff efficiency.

After the FA procedure, the pairwise comparison matrix is developed individually under each component of resilience (Table 5) during AHP analysis. By repeating the AHP procedure, the subjective weights of the four components in the first level are then derived according to opinions of local government (Table 6). Consequently, the weights of factors in the second level and those of components in the first level are shown in Table 7. Because all CR values are less than 0.1, all subjective weights assigned by experts are accepted from a statistical perspective.

**Table 5:** Pairwise comparison matrix: Extracted factors in second level.

Component	Social		Economic		Infrastructural		Natural		
**Factor**	Demographics	Community preparedness/ service	Industry and asset exposure	Economic stability and commercial infrastructure exposure	Response rapidness and capacity	Sheltering access and capacity	Elevation and slope	Strong storm risk and landslide risk	Flooding risk
Demographics	—	1/2							
Community preparedness /service	2	—							
Industry and asset exposure			—	1					
Economic stability and commercial infrastructure exposure			1	—					
Response rapidness and capacity					—	3/2			
Sheltering access and capacity					2/3	—			
Elevation and slope							—	1/3	1/5
Strong storm risk and landslide risk							3	—	1/2
Flooding risk							5	2	—

**Note**: Each cell in the matrix corresponds to a pairwise comparison of factors. For example, under the social dimension, the index of community preparedness/service is considered twice as important as that of demographics, and thus the cell between the two indices is assigned 2 and the corresponding cell on the other side of the diagonal is assigned 1/2. Since it does not make sense to compare a factor to itself, the diagonal elements of the matrix are irrelevant and thus assigned null values.

**Table 6:** Pairwise comparison matrix: Four components of resilience in first level.

**Component**	**Social**	**Economic**	**Infrastructural**	**Natural**
**Social**	—	1	1/3	1/5
**Economic**	1	—	2/3	2/5
**Infrastructural**	3	3/2	—	1/2
**Natural**	5	5/2	2	—

**Table 7:** Relative weights of factors in second level and components in first level.

Goal	Level 1	Level 2	Weight	CR
**A composite index of resilience**	Social (0.11)	Demographics	0.333	—
		Community preparedness /service	0.667	
	Economic (0.155)	Industry and asset exposure	0.500	—
		Economic stability and commercial infrastructure exposure	0.500	
	Infrastructure (0.261)	Response rapidness and capacity	0.600	—
		Sheltering access and capacity	0.400	
	Natural (0.475)	Elevation and slope	0.109	0.003
		Strong storm risk and landslide risk	0.309	
		Flooding risk	0.582	
**Consistency Ratio (CR)**	0.024			

### Geographic variations of resilience

The focus of resilience measurement research is to explore whether the analysis units are disproportionately affected by a damaging event due to differences in factors related to the social, economic, infrastructural, and natural components of resilience. To effectively reduce the possible disaster damage and accelerate the recovery process, local government and planners should understand (1) the dominant site-specific factors that give rise to disaster resilience within the area they manage and (2) the spatial pattern of resilience across their regions. Addressing these two questions could help local governments and planners to identify sites that might be more vulnerable to disaster damage or would likely have a poor adaptive capacity to response and recovery after disasters. The results of FA and AHP analysis help to answer the first question and suggest that although the whole Nansha district is exposed to typhoon disasters, geographic variation exists among different sites because of spatial heterogeneities in social, economic, infrastructural, and natural factors.

**Fig 8:**
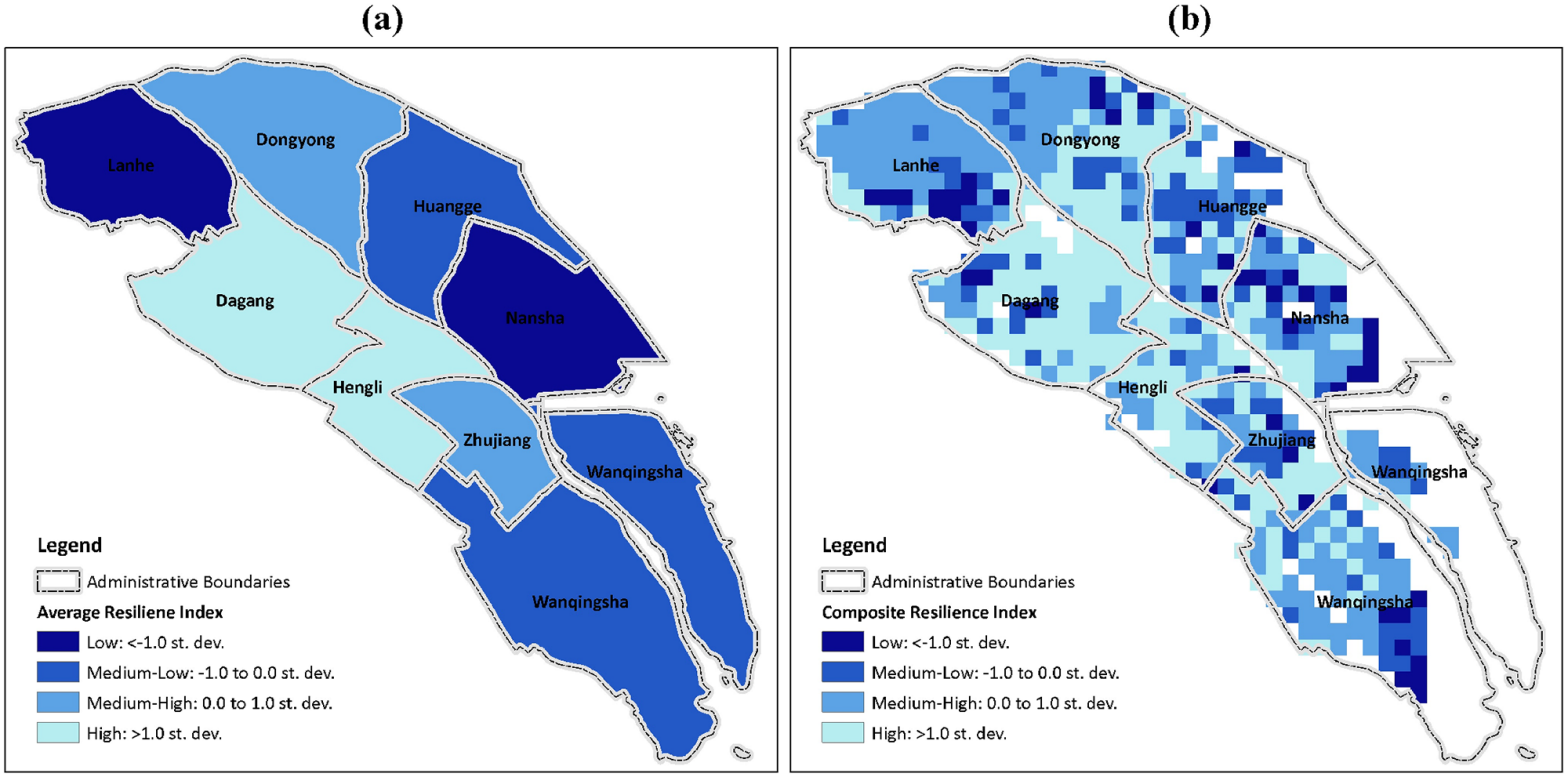
Composite index of resilience to typhoon disaster in Nansha district. a) sub-district level; b) 1×1-km grid level (empty grids represent grids with zero population). The map was generated using the free and open source software QGIS version 2.18 (http://www.qgis.org/en/site/about/index.html).

**Fig 9:**
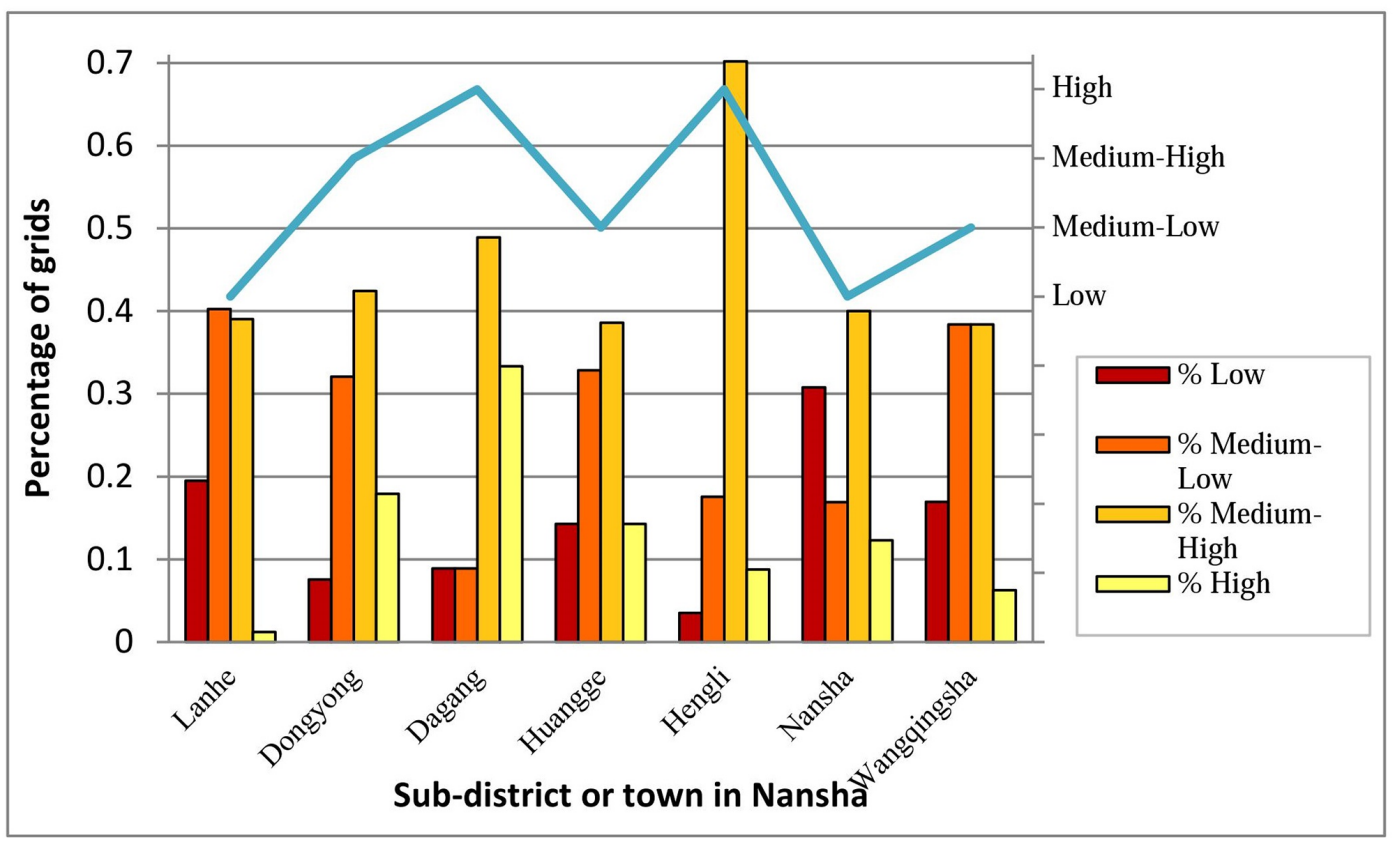
Percentage of grids in sub-districts with z-scores in different resilience classes and average class of each sub-district.

By mapping the calculated composite resilience index, local governments and planners could detect potential hot spots or cold spots of disaster resilience and then further explore at a particular location the dominant components, factors, and variables at the bottom level hierarchically. In this study, a map of 1×1-km grid cells classified by the z-scores of the composite index allows one to quickly identify the hotspots within a sub-district or town (Fig 8a) rather than simply evaluate the resilience of sub-districts or towns on the basis of average scores as a whole (Fig 8b). Just like most resilience maps at the administrative level or broad census unit level, Fig 8b could not fully capture the resilience variation across an administrative unit. For further explanation, we define high resilience class with transformed z-scores of resilience index greater than 1.0 (i.e., greater than one standard deviation from the mean of analysis unit), medium to high resilience class with z-scores between 0 to 1.0, medium to low resilience class with z-scores between −1.0 to 0.0, and low resilience class with z-scores smaller than −1.0. We then calculate the percentage of grids in sub-districts with z-scores in different resilience classes and finally generate Fig 9 for comparison among different sub-districts (or towns). Accordingly, with regard to average resilience scores, Dongyong is considered to be in a medium to high class, whereas Dagang is considered to be in a high resilience class. However, based on the percentage of grids in each resilience class within the two towns, we could see that the percentage of grids with a low resilience score is greater in Dagang than in Dongyong. Therefore, the average resilience score of a sub-district or town as a whole would not make sense for detecting potential hot spots or cold spots in a particular location within a sub-district or town. However, a map of the grid-based resilience score could narrow this gap and help local governments and urban planners to design site-specific measures in small-scale projects.

**Fig 10:**
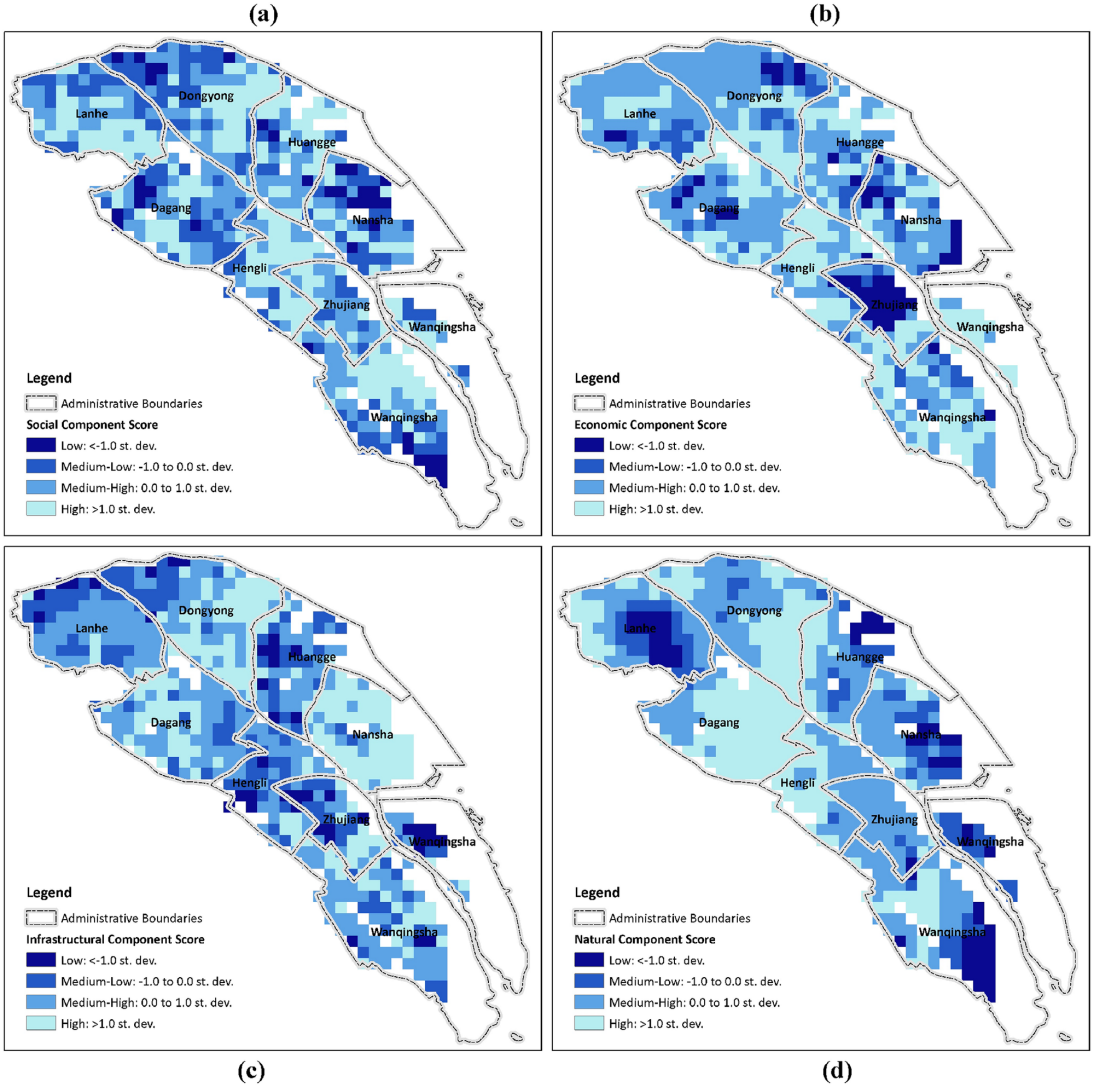
Maps of resilience component score. a) social component; b) economic component; c) infrastructural component; d) natural component; classified as low (< −1.0 SD), medium-low (−1.0 to 0.0 SD), medium-high (0.0 to 1.0 SD), and high (>1.0 SD). The map was generated using the free and open source software QGIS version 2.18 (http://www.qgis.org/en/site/about/index.html).

In contrast, mapping the components of resilience could assist local governments and urban planners to identify site-specific dominant factors (Fig 10). Using the two sub-districts in low resilience class as examples, hot spots of low resilience in Lanhe are mostly gathered around the southwest corner due to the poor natural condition of resilience and are likely aggravated by irrational economic planning and imperfect infrastructure construction, whereas the dominant factors in Nansha sub-district are mostly related to natural and socioeconomic factors rather than to infrastructural ones. In addition, from this comparison, it is worth noting that natural factors play important roles in determining the resilience class of both sub-districts. As two critical variables that represent natural resilience, the predicted typhoon wind speed and precipitation in a high spatial resolution could support the need for local governments and planners to determine site-specific measures when developing risk-reduction strategies and not to implement generic strategies with the assumption that the whole sub-district or town has similar exposure to strong storm and flooding risk.

## Summary and conclusions

The concept of typhoon resilience has introduced a new philosophy to urban systems: “living with typhoons.” It should be most noteworthy for mapping the resilience indices of typhoon-prone areas, such as the coastal regions in Nansha, as a substitute tool for local governments, especially decision makers. In this study, we have therefore developed a hierarchical structure of indicators for resilience assessment that consists of indicators from different dimensions of resilience (i.e., social, economic, infrastructural, and natural) at the first level, a set of sub-factors used to describe the four dimensions at the second level, and more detailed indicators at the third level. Following the hierarchical structure, this study aggregated a set of indicators with a coupled FA and AHP approach and incorporated them into the typhoon resilience evaluation process. During the assessment procedure, the values for each indicator were calculated, and the final resilience assessment result was mapped with the use of several techniques, such as WRF for modeling the wind speeds and precipitation caused by typhoons, R software for predicting the typhoons’ long-term return level, and GIS for visualizing typhoon resilience in the study area.

The results of this study demonstrate that resilience to typhoon disasters differs across the study area and that the resilience level and scores of its subcomponents (e.g., social, economic, infrastructural, and natural components) were not consistent either among or within sub-districts (or towns). The methods used here contribute to the melding of research on meteorology modeling and extreme value analysis with the development of composite resilience indicators. Using the WRF model to retrieve each typhoon track to simulate and extract the possible wind and precipitation levels in each grid square (1 × 1 km) greatly improved the resolution of data compared with traditional studies, whereas extreme value analysis of typhoons rather than direct use of raw historical data made a theoretical basement for local governments and urban planners to make site-specific decisions on typhoon preparation and recovery plans. Using a hierarchical framework that incorporated FA and AHP for resilience assessment made it possible to construct a composite resilience index from statistical, theoretical, and practical perspectives.

Because this is one of the first such attempts aimed at measuring resilience at a high spatial resolution and combining simulation, statistical analysis, and composite indicator studies, this study indicates areas of opportunity. Our first recommendation is that the modeling outcomes of typhoon disasters could be further improved if greater computing resources could be secured. Second, because Nansha is a newly established district of Guangzhou in Guangdong Province with a shortage of attributes of institutional systems, such as governance, policy, and management, institutional variables are not included within this framework. If data are available, the effects of institutional components could be considered in further studies.

## Supporting information

S1 Text Configuration and evaluation of the WRF model. (DOCX)

S2 Text Explanations about the software used for of each figure. (DOCX)

S1 Fig Three nested domains (9km outer, 3km middle and 1km inner) centered over the Nansha district for the implementation of WRF model. The map was generated using the free and open source software NCAR Command Language version 6.4.0 (2017) (http://dx.doi.org/10.5065/D6WD3XH5).
(TIF)

S2 Fig Modeled and observed typhoon tracks of Usagi. Two tracks are both in 3-hour interval; the modeled track is in red and the observed in black. The map was generated using the free and open source software NCAR Command Language version 6.4.0 (2017) (http://dx.doi.org/10.5065/D6WD3XH5).
(TIF)

S3 Fig Time dependence of maximum surface wind speed. The black line indicates the simulated data, and the red line indicates the observations. The map was generated using the free and open source software NCAR Command Language version 6.4.0 (2017) (http://dx.doi.org/10.5065/D6WD3XH5).
(TIF)

S4 Fig The 24-hour accumulated precipitation (mm) of Typhoon Usagi. Observations (left) and simulated data (right); the duration is from 0000 UTC 22 September to 0000 UTC 23 September 2013. The map was generated using the free and open source software NCAR Command Language version 6.4.0 (2017) (http://dx.doi.org/10.5065/D6WD3XH5).
(TIF)

S5 Fig The location of the sample grid. The map was generated using the free and open source software QGIS version 2.18 (http://www.qgis.org/en/site/about/index.html).
(TIF)

## References

[1] Rose A., Defining and measuring economic resilience to disasters. Disaster Prevention and Management: An International Journal, 2004 13(4): p. 307–314.

[2] Boon H.J., Disaster resilience in a flood-impacted rural Australian town. Natural hazards, 2014 71(1): p. 683–701.

[3] United Nations Office for Disaster Risk Reduction (UNISDR), 18060066. National Science and Technology Council, Committee on Environment and Natural Resources, 2005. http://www.sdr.gov/docs/GrandChallengesSecondPrinting.pdf.

[4] UNISDR, *How to Make Cities More Resilient—A Handbook for Mayors and Local Government Leaders*. Geneva, Switzerland: United Nations International Strategy for Disaster Reduction, 2012 http://www.unisdr.org/files/26462_handbookfinalonlineversion.pdf.

[5] Prasad N., et al, *Climate resilient cities*: *A primer on reducing vulnerabilities to disasters*. 2009: Washington, DC: World Bank.

[6] Richard B., Kate B., Cynthia P., Charlotte B., Siena C., & Christopher H., *Resilience Cities*: *A Grosvenor research report*. The Grosvenor group in Canada, 2014 http://www.grosvenor.com/getattachment/194bb2f9-d778-4701-a0ed-5cb451044ab1/ResilientCitiesResearchReport.pdf

[7] Cutter S.L., Burton C.G., and Emrich C.T., Disaster resilience indicators for benchmarking baseline conditions. Journal of Homeland Security and Emergency Management, 2010 7(1).

[8] Meerow S., Newell J.P., and Stults M., Defining urban resilience: A review. Landscape and urban planning, 2016 147: p. 38–49.

[9] Timmerman P., *Vulnerability resilience and collapse ofsociety* A ReviewofModels and Possible Climatic Appli-cations. Toronto, Canada Institute for Environmental Studies, University of Toronto, 1981.

[10] Dovers S.R. and Handmer J.W., Uncertainty, sustainability and change. Global Environmental Change, 1992 2(4): p. 262–276.

[11] Blaikie P., et al, *At risk*: *natural hazards*, *people’s vulnerability and disasters*. 2014: Routledge.

[12] Gunderson L.H., Ecological resilience—in theory and application. Annual review of ecology and systematics, 2000 31(1): p. 425–439.

[13] Berke P.R. and Campanella T.J., Planning for postdisaster resiliency. The Annals of the American Academy of Political and Social Science, 2006 604(1): p. 192–207.

[14] Council N.R., *Facing hazards and disasters*: *Understanding human dimensions*. 2006: National Academies Press.

[15] Paton D. and Johnston D.M., *Disaster resilience*: *an integrated approach*. 2006: Charles C Thomas Publisher.

[16] Norris F.H., et al, Community resilience as a metaphor, theory, set of capacities, and strategy for disaster readiness. American journal of community psychology, 2008 41(1–2): p. 127–150. doi: 10.1007/s10464-007-9156-6 18157631 10.1007/s10464-007-9156-6

[17] Yu S., et al, Quantitative assessment of disaster resilience: An empirical study on the importance of post-disaster recovery costs. Reliability Engineering & System Safety, 2015 137: p. 6–17.

[18] Song J., Huang B., and Li R., Measuring Recovery to Build up Metrics of Flood Resilience Based on Pollutant Discharge Data: A Case Study in East China. Water, 2017 9(8): p. 619.

[19] Adger W.N., et al, Social-ecological resilience to coastal disasters. Science, 2005 309(5737): p. 1036–1039. doi: 10.1126/science.1112122 16099974 10.1126/science.1112122

[20] Cutter, S.L. and H. Director, A framework for measuring coastal hazard resilience in New Jersey communities. White Paper for the Urban Coast Institute, 2008.

[21] Cutter S.L., Ash K.D., and Emrich C.T., The geographies of community disaster resilience. Global environmental change, 2014 29: p. 65–77.

[22] Babbie E.R., *The basics of social research*. 2013: Cengage Learning.

[23] Mayunga J.S., *Measuring the measure*: *A multi-dimensional scale model to measure community disaster resilience in the US Gulf Coast region*. 2009, Texas A&M University.

[24] Klein R.J. and Nicholls R.J., Assessment of coastal vulnerability to climate change. Ambio, 1999: p. 182–187.

[25] Bruneau M., et al, A framework to quantitatively assess and enhance the seismic resilience of communities. Earthquake spectra, 2003 19(4): p. 733–752.

[26] Cutter S.L., Boruff B.J., and Shirley W.L., Social vulnerability to environmental hazards. Social science quarterly, 2003 84(2): p. 242–261.

[27] Jordan E. and Javernick-Will A., Indicators of community recovery: content analysis and Delphi approach. Natural hazards review, 2013 14(1): p. 21–28.

[28] Lam N.S.-N., et al, Assessment of vulnerability and adaptive capacity to coastal hazards in the Caribbean Region. Journal of Coastal Research, 2014 70(sp1): p. 473–478.

[29] Cutter S.L., et al, A place-based model for understanding community resilience to natural disasters. Global environmental change, 2008 18(4): p. 598–606.

[30] Joerin J. and Shaw R., *Chapter 3 mapping climate and disaster resilience in cities*, *in Climate and disaster resilience in cities*. 2011, Emerald Group Publishing Limited p. 47–61.

[31] Reams M.A., Lam N.S., and Baker A., Measuring capacity for resilience among coastal counties of the US Northern Gulf of Mexico Region. American journal of climate change, 2012 1(4): p. 194 doi: 10.4236/ajcc.2012.14016 27500076 10.4236/ajcc.2012.14016 PMC4972028

[32] Stevens M.R., Berke P.R., and Song Y., Creating disaster-resilient communities: Evaluating the promise and performance of new urbanism. Landscape and Urban Planning, 2010 94(2): p. 105–115.

[33] o’Brien K., et al, Mapping vulnerability to multiple stressors: climate change and globalization in India. Global environmental change, 2004 14(4): p. 303–313.

[34] Schröter D., et al, Ecosystem service supply and vulnerability to global change in Europe. science, 2005 310(5752): p. 1333–1337. doi: 10.1126/science.1115233 16254151 10.1126/science.1115233

[35] The Planning Research Center of Nansha District, The Comprehesive Planning of Nansha New City. 2012: http://www.gzns.gov.cn/zwxxgk/ghjh/201709/P020170928290188734529.pdf (In Chinese).

[36] Gilleland E. and Katz R.W., New software to analyze how extremes change over time. Eos, Transactions American Geophysical Union, 2011 92(2): p. 13–14.

[37] Davison A.C. and Smith R.L., Models for exceedances over high thresholds. Journal of the Royal Statistical Society. Series B (Methodological), 1990: p. 393–442.

[38] Hosking J.R. and Wallis J.R., Parameter and quantile estimation for the generalized Pareto distribution. Technometrics, 1987 29(3): p. 339–349.

[39] Palutikof J., et al, A review of methods to calculate extreme wind speeds. Meteorological applications, 1999 6(2): p. 119–132.

[40] Wilks D.S., *Statistical methods in the atmospheric sciences*. Vol. 100 2011: Academic press.

[41] Gilleland E. and Katz R.W., Extremes 2.0: an extreme value analysis package in r. submitted to journal of statistical software, 2014.

[42] Shahid S. and Behrawan H., Drought risk assessment in the western part of Bangladesh. Natural Hazards, 2008 46(3): p. 391–413.

[43] Bravo G. and Potvin L., Estimating the reliability of continuous measures with Cronbach’s alpha or the intraclass correlation coefficient: toward the integration of two traditions. Journal of clinical epidemiology, 1991 44(4–5): p. 381–390. 2010781 10.1016/0895-4356(91)90076-l

[44] Cumming G.S., et al, An exploratory framework for the empirical measurement of resilience. Ecosystems, 2005 8(8): p. 975–987.

[45] U.S. Indian Ocean Tsunami Warning System Program, *How resilient is your coastal community*? *A guide for evaluating coastal community resilience to tsunamis and other coastal hazards*. 2007: Bangkok, Thailand: U.S. Agency for International Development http://www.crc.uri.edu/download/CCRGuide_lowres.pdf.

[46] Li X., et al, Measuring county resilience after the 2008 Wenchuan earthquake. Natural Hazards and Earth System Sciences Discussions, 2015 3: p. 81–122.

[47] Fu J, J.D., Huang Y, 1 KM Grid Population Dataset of China. Global Change Research Data Publishing & Repository, 2014. http://www.geodoi.ac.cn/weben/doi.aspx?Id=131.

[48] Schneiderbauer S., Ehrlich D., and Birkmann J., Social levels and hazard (in) dependence in determining vulnerability. Measuring vulnerability to natural hazards: Towards disaster resilient societies, 2006: p. 78–102.

[49] Burton C.G., A validation of metrics for community resilience to natural hazards and disasters using the recovery from Hurricane Katrina as a case study. Annals of the Association of American Geographers, 2015 105(1): p. 67–86.

[50] Jones B. and Andrey J., Vulnerability index construction: methodological choices and their influence on identifying vulnerable neighbourhoods. International journal of emergency management, 2007 4(2): p. 269–295.

[51] Wood N.J., Burton C.G., and Cutter S.L., Community variations in social vulnerability to Cascadia-related tsunamis in the US Pacific Northwest. Natural Hazards, 2010 52(2): p. 369–389.

[52] Saaty R.W., The analytic hierarchy process—what it is and how it is used. Mathematical modelling, 1987 9(3–5): p. 161–176.

[53] Ernstson H., et al, Urban transitions: on urban resilience and human-dominated ecosystems. AMBIO: A Journal of the Human Environment, 2010 39(8): p. 531–545. 10.1007/s13280-010-0081-9 PMC3357675 21141773

[54] Cutter S.L., The landscape of disaster resilience indicators in the USA. Natural Hazards, 2016 80(2): p. 741–758.

[55] Wang S.-H., Huang S.-L., and Budd W.W., Resilience analysis of the interaction of between typhoons and land use change. Landscape and Urban Planning, 2012 106(4): p. 303–315.

