# Supplementary material for: Assessing local resilience to typhoon disasters: A case study in Nansha, Guangzhou
Source: PLoS One. 2018 Mar 9;13(3):e0190701. doi: 10.1371/journal.pone.0190701 (PMC5844519; doi:10.1371/journal.pone.0190701)
Supplement: S1 Text — (DOCX) [file pone.0190701.s001.docx]

**S1 Text. Configuration and evaluation of the WRF model**

With respect to the duration of each typhoon sample over the study region, experiments were integrated for 2 to 4 days and three nested domains (9km outer, 3km middle and 1km inner) were introduced to the model with the corresponding time steps of 30s, 10s and 3.33s, respectively (**S1 Fig**). The major physical schemes of the WRF model involved the Lin microphysics scheme, the Rapid Radiative Transfer Model for shortwave and longwave radiation scheme, the National Centers for Environmental Prediction, Oregon State University, Air Force, and Hydrologic Research Laboratory’s land surface module, and the Yonsei University planetary boundary layer scheme. The boundary conditions and initial field were derived from the National Centers for Environmental Prediction Final (NCEP/FNL) 1-degree global tropospheric analysis dataset (https://rda.ucar.edu/datasets/ds083.2/). The first 12 hours were treated as the spin-up period.

The WRF-simulated typhoon track, maximum surface wind speed, and 24-hour accumulated precipitation of each typhoon sample were compared with observed records. The observed typhoon tracks and wind speed data were collected from the China Meteorological Administration (CMA) Tropical Cyclone Best Track Dataset (http://tcdata.typhoon.org.cn/en/zjljsjj_zlhq.html). The 24-hour accumulated precipitation data were obtained from an automatic meteorological station in Guangdong Province. The comparison results of Typhoon Usagi are shown as examples in **S2—S4** **Fig**. Accordingly, **S2** **Fig** shows that the modeled typhoon track follows and approaches closely the observed one during the simulation period. Although the simulated maximum wind speed is lower than the observed due to the coarse resolution of the NCEP/FNL initial field and other model parameters, the tendency is consistent with that of the observed records (**S3** **Fig**). **S4** **Fig** shows the 24-hour precipitation from 0000 UTC 22 September to 0000 UTC 23 September 2013 from the observed data and modeled data. The magnitude and spatial distribution of the simulated precipitation both fit well with the observed records. As a whole, the model reproduced well the typhoon tracks, wind speeds and precipitation data.
