# Supplementary material for: Assessing local resilience to typhoon disasters: A case study in Nansha, Guangzhou
Source: PLoS One. 2018 Mar 9;13(3):e0190701. doi: 10.1371/journal.pone.0190701 (PMC5844519; doi:10.1371/journal.pone.0190701)
Supplement: S2 Text — (DOCX) [file pone.0190701.s002.docx]

**S6 Text. Explanations about the software used for of each figure.**

The software used for each figure in our manuscript are listed as follows:

- **Figs 1**, **3**, **6**, **8**, **10**, **S5** were created using the free and open source software ***QGIS*** [1] ***version 2.18*** (http://www.qgis.org/en/site/about/index.html).
- **Fig 2** and **Figs S1-S4** were created using ***NCAR Command Language (NCL)*** [2] which serves as an interpreted language designed specifically for scientific data analysis and visualization. It is **free of charge**.
- **Fig 4** and **Fig 7** were produced using ***R*** which serves as a language and environment for statistical computing and graphics and is **free of charge**.
- **Fig 5** and **Fig 9** were created using **licensed** ***Microsoft Office Visio 2016*** and ***Microsoft Office Excel 2016***, respectively. Our department has their licenses legally.

**Reference:**

[1] QGIS Development Team, 2009. QGIS Geographic Information System. Open Source Geospatial Foundation. URL http://qgis.osgeo.org

[2] The NCAR Command Language (Version 6.4.0) [Software]. (2017). Boulder, Colorado: UCAR/NCAR/CISL/TDD. http://dx.doi.org/10.5065/D6WD3XH5
