# Supplementary figures and images for: Assessing local resilience to typhoon disasters: A case study in Nansha, Guangzhou
Source: PLoS One. 2018 Mar 9;13(3):e0190701. doi: 10.1371/journal.pone.0190701 (PMC5844519; doi:10.1371/journal.pone.0190701)

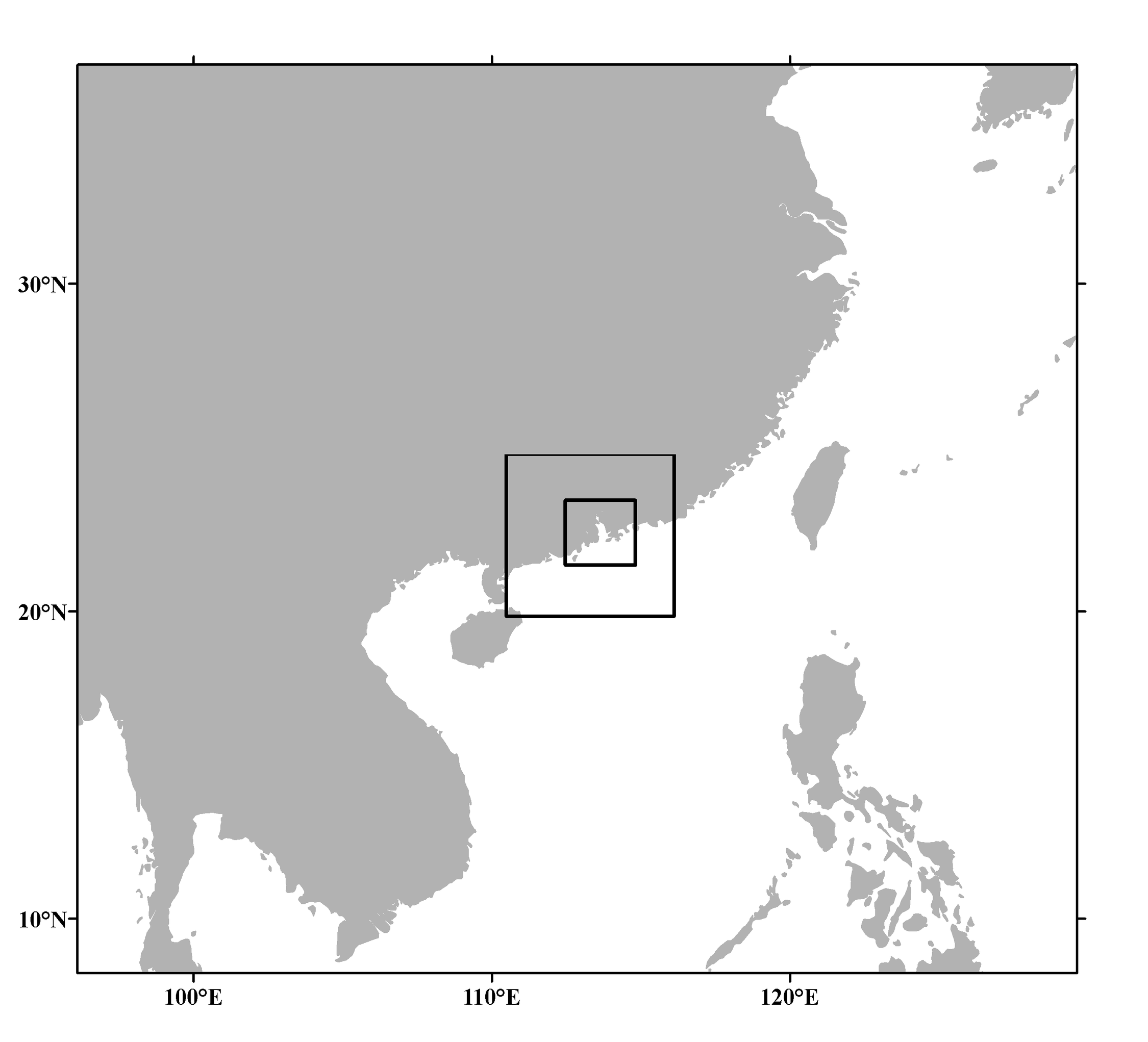

Supplement: S1 Fig — The map was generated using the free and open source software NCAR Command Language version 6.4.0 (2017) (http://dx.doi.org/10.5065/D6WD3XH5). (TIF) [file pone.0190701.s003.tif]

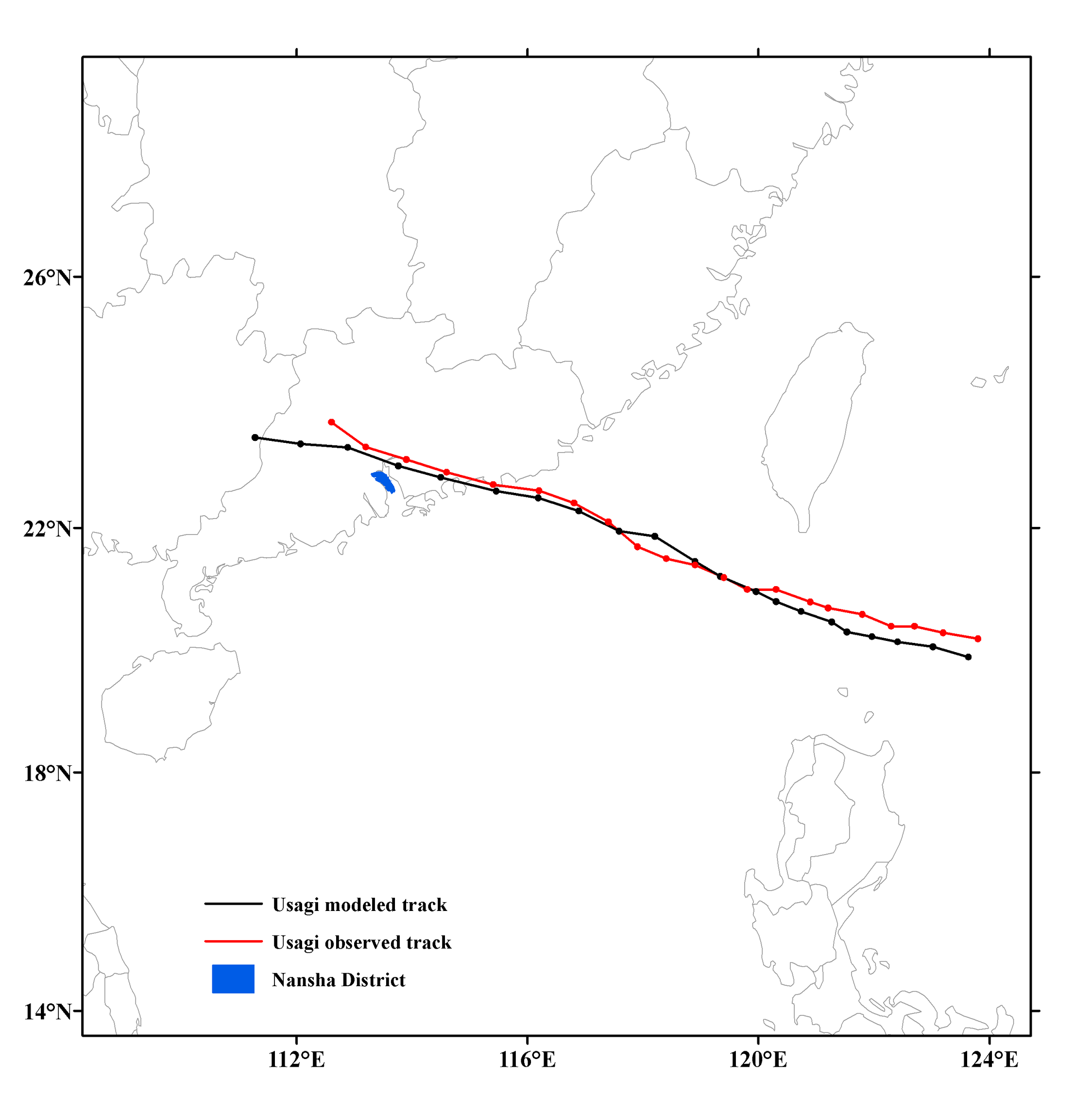

Supplement: S2 Fig — Two tracks are both in 3-hour interval; the modeled track is in red and the observed in black. The map was generated using the free and open source software NCAR Command Language version 6.4.0 (2017) (http://dx.doi.org/10.5065/D6WD3XH5). (TIF) [file pone.0190701.s004.tif]

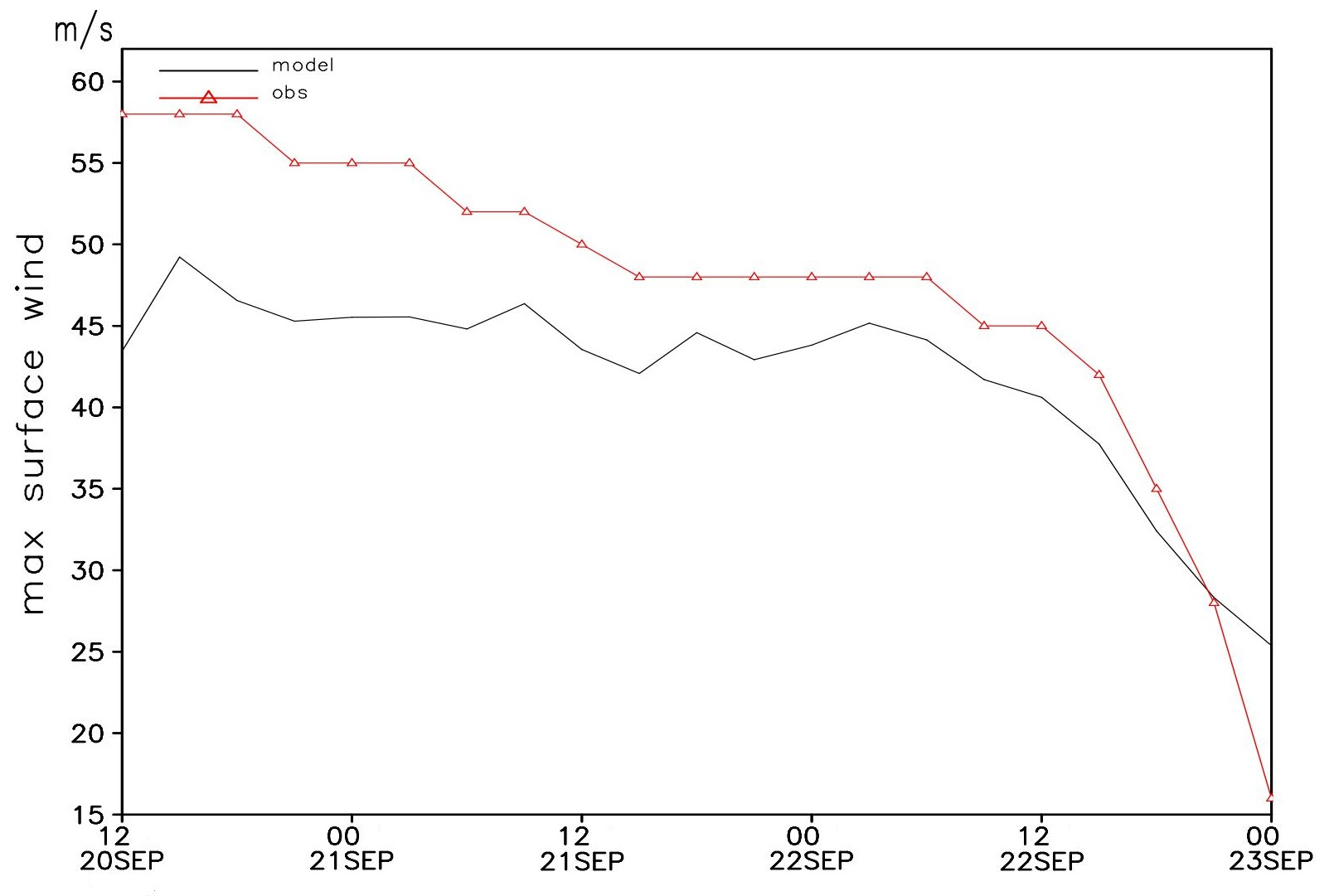

Supplement: S3 Fig — The black line indicates the simulated data, and the red line indicates the observations. The map was generated using the free and open source software NCAR Command Language version 6.4.0 (2017) (http://dx.doi.org/10.5065/D6WD3XH5). (TIF) [file pone.0190701.s005.tif]

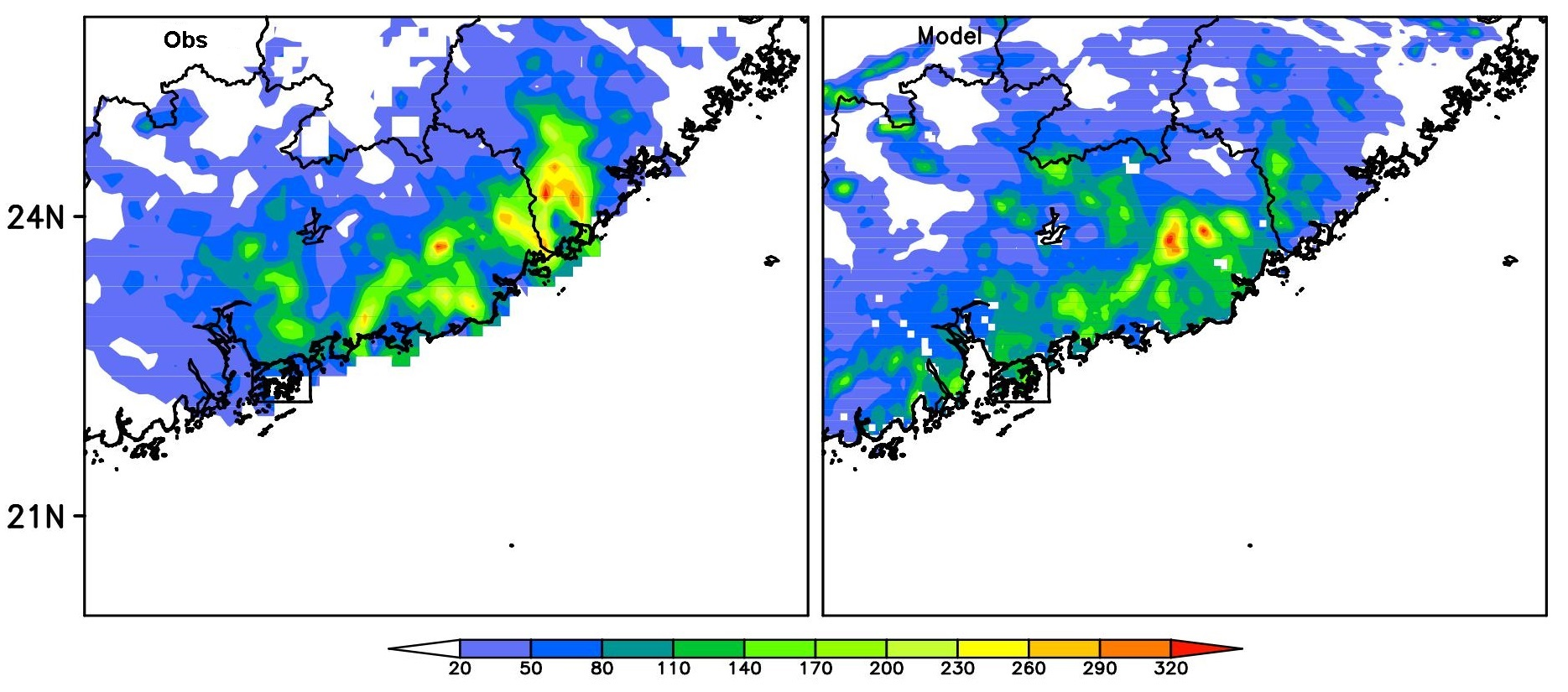

Supplement: S4 Fig — Observations (left) and simulated data (right); the duration is from 0000 UTC 22 September to 0000 UTC 23 September 2013. The map was generated using the free and open source software NCAR Command Language version 6.4.0 (2017) (http://dx.doi.org/10.5065/D6WD3XH5). (TIF) [file pone.0190701.s006.tif]

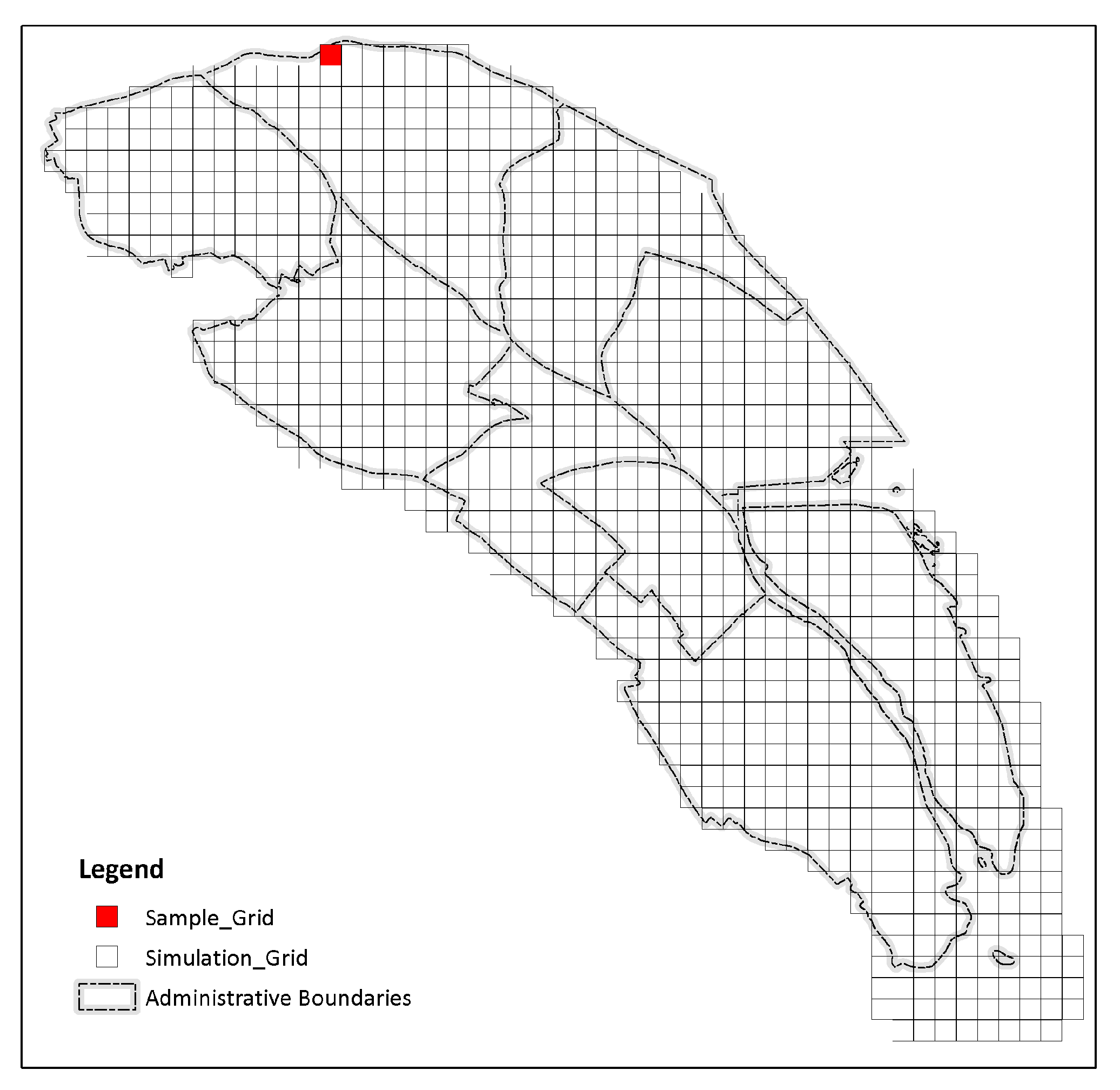

Supplement: S5 Fig — The map was generated using the free and open source software QGIS version 2.18 (http://www.qgis.org/en/site/about/index.html). (TIF) [file pone.0190701.s007.tif]
